# Divergent Roles of mGlu2 and mGlu3 Receptors in Amyloid‐β Production and Cognitive Dysfunctions in Alzheimer's Disease

**DOI:** 10.1002/advs.202523791

**Published:** 2026-04-14

**Authors:** Pierre‐André Lafon, Mireille Elodie Tsitokana, Ugo Guy Alenda, Yen‐Ling Lian, Clémentine Eva Philibert, Mathieu Oosterlaken, Marta Cimadevila, Gaëlle Dudon, Jessica Monnic, Salomé Roux, Julie Bessié, Séverine Diem, Franck Vandermoere, Laurent Prézeau, Patrick Chames, Julie Kniazeff, Sylvie Claeysen, Anaïs Menny, Jean‐Philippe Pin, Véronique Perrier, Jianfeng Liu, Philippe Rondard

**Affiliations:** ^1^ Cellular Signaling Laboratory International Research Center for Sensory Biology and Technology of MOST Key Laboratory of Molecular Biophysics of MOE, and College of Life Science and Technology Huazhong University of Science and Technology Wuhan China; ^2^ Institut de Génomique Fonctionnelle (IGF) University of Montpellier, CNRS, INSERM Montpellier Cedex 5 France; ^3^ Institut des Neurosciences de Montpellier (INM) University of Montpellier, INSERM, CNRS Montpellier France; ^4^ Aix Marseille University, CNRS, INSERM, Institut Paoli‐Calmettes, CRCM Marseille France; ^5^ Bioland Laboratory Guangzhou Regenerative Medicine and Health Guangdong Laboratory Guangzhou China; ^6^ Hubei Jiangxia Laboratory Wuhan Hubei China

**Keywords:** G protein‐coupled receptor, nanobody, single domain camelid antibody, time‐resolved fluorescence energy transfer, VHH

## Abstract

Immunotherapy is a promising avenue for reducing amyloid‐β (Aβ) accumulation, a hallmark of Alzheimer's disease (AD) pathology. Camelid single domain antibodies, called nanobodies, offer several advantages over conventional monoclonal antibodies, including improved brain penetration and fine‐tuning of the targeted neuroreceptors, and may represent an effective strategy to modulate Aβ production. Among potential therapeutic targets, group II metabotropic glutamate receptors (mGluR2 and mGluR3) have been implicated in Aβ regulation, though their individual contributions remain unclear. Here, we showed that activation of mGluR2 significantly increases Aβ peptides and sAPPβ production in a cellular model, by enhancing the internalization of amyloid precursor protein (APP) and its subsequent amyloidogenic processing. In contrast, mGluR3 directly interacts with APP, protecting it from amyloidogenic cleavage and favoring its non‐amyloidogenic processing. We used a brain‐penetrant nanobody acting as a selective positive allosteric modulator of mGluR2 to validate its role in Aβ dynamics in vivo. Chronic administration of this nanobody in 5xFAD mice accelerated amyloid plaque deposition and worsened cognitive deficits. These findings establish mGluR2 as a target in AD and demonstrate that its selective modulation by nanobodies influences Aβ pathology. This also highlights the potential of nanobodies as next‐generation therapeutic agents for modulating neuroreceptors activity in AD.

## Graphical abstract

This study explores the opposing effects of the mGluR2 and mGluR3 receptors on amyloid precursor protein processing. mGluR2 promotes amyloidogenic cleavage, while mGluR3 favors non‐amyloidogenic pathways. Using a brain‐penetrant nanobody as a mGluR2 positive allosteric modulator, the study uncovers how its chronic activation aggravates amyloid‐β burden and cognitive deficits, positioning nanobodies as powerful tools to influence receptor‐driven amyloid‐β regulation.

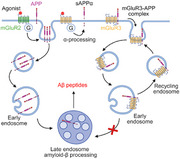

## 1 Introduction

Alzheimer's disease (AD) is the most prevalent neurodegenerative disorder and the leading cause of dementia worldwide [1]. It is characterized by progressive cognitive decline, memory impairments, and the accumulation of pathological protein aggregates, including extracellular amyloid plaques and intracellular neurofibrillary tangles [2, 3, 4]. Amyloid plaques are primarily composed of amyloid‐β (Aβ) peptides [5], generated via sequential cleavage of the amyloid precursor protein (APP) by β‐secretase (BACE1) and γ‐secretase [6]. Despite extensive efforts, most small‐molecule therapies targeting these pathological pathways have failed to show meaningful clinical efficacy [7]. Monoclonal antibodies directed against Aβ peptides have recently received FDA approval for a subgroup of patients in the early stages of AD with mild cognitive impairments [8, 9, 10, 11]. However, the approval of aducanumab was controversial [12], as repeated high‐dose administrations triggered the development of autoantibodies and amyloid‐related imaging abnormalities (ARIA) in up to 43% of treated patients [13]. While antibody‐based therapy holds promise, there is an urgent need to identify new targets capable of reducing Aβ load in patients’ brains, enabling the development of next‐generation antibodies with improved safety profiles.

Among emerging targets, group II metabotropic glutamate receptors (mGluR2 and mGluR3) [14], G protein‐coupled receptors (GPCRs), have gained attention due to their involvement in synaptic regulation and potential roles in AD progression [15]. Although both receptors are structurally similar [16], their individual contributions to Aβ dynamics remain unclear. For example, selective activation of mGluR2 with LY566332 has been reported to exacerbate Aβ toxicity in cocultures of neurons and astrocytes, whereas non‐selective activation of mGluR2 and mGluR3 with LY379268 appears to provide neuroprotective effects [17]. Additionally, activation of group II mGluRs has been shown to increase Aβ_1‐42_ secretion in synaptosomes from an AD mouse model [18]. Thus, clarifying the specific roles of mGluR2 and mGluR3 is essential for therapeutic development, yet selective pharmacological tools remain limited.

Camelid single‐domain antibodies (nanobodies or V_HH_) [19] have recently emerged as promising agents for selectively targeting and modulating the activity of GPCRs. Their small size (∼15 kDa), high stability, low immunogenicity [20], and ability to penetrate the brain make them attractive candidates for the treatment of neurological diseases [21, 22, 23]. Our group has developed nanobodies with subtype‐specific affinity for mGluRs, functioning as either agonists or positive allosteric modulators (PAMs) [22, 24, 25, 26]. Among these, DN13‐DN1 is a brain‐penetrant biparatopic nanobody that acts as a PAM specific to mGluR2 [22]. It can be detected in brain tissue up to seven days after a single intraperitoneal injection and has been shown to rescue cognitive deficits in mouse models recapitulating schizophrenia symptoms [22], as mGluR2 is involved in this mental disorder [27]. All these DN13‐DN1 properties make it a valuable tool for investigating mGluR2 function in AD.

In the present study, we investigated the effects of mGluR2 activation, either with small molecules or via DN13‐DN1, on Aβ production. Using transfected cell models, we demonstrate that mGluR2 activation enhances Aβ_1‐42_ and sAPPβ generation and secretion, a process linked to increased internalization of APP and its subsequent cleavage by β‐secretase. In contrast, mGluR3 interacts directly with APP, protecting it from amyloidogenic processing and preventing Aβ production, rerouting APP trafficking towards recycling endosomes. Activation of mGluR3 promotes sAPPα production, favoring non‐amyloidogenic APP processing. Furthermore, chronic administration of DN13‐DN1 in 5xFAD transgenic mice exacerbated cognitive impairments and accelerated amyloid deposition. These findings provide compelling evidence that mGluR2 plays a deleterious role in AD progression and establish a proof‐of‐concept that nanobody‐mediated modulation of this receptor can influence both Aβ burden and behavioral outcomes. These findings further highlight the therapeutic potential of nanobodies as precision tools to modulate mGluR activity and AD progression.

## 2 Results

### 2.1 Activation of mGluR2, but not mGluR3, Increases Aβ_1‐42_ Production

**FIGURE 1:**
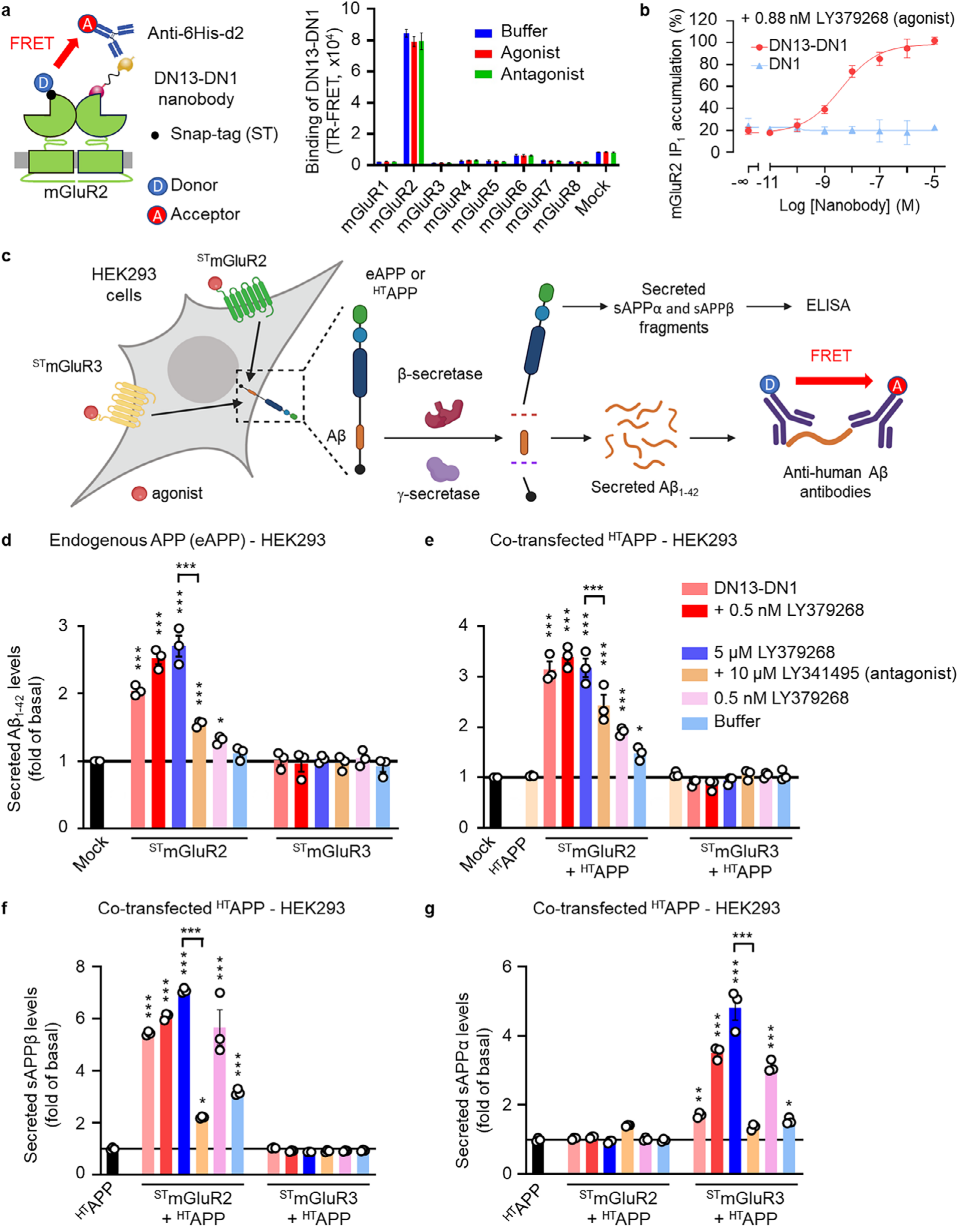
mGluR2 activation increases Aβ_1‐42_ and sAPPβ secretion, while mGluR3 promotes sAPPα secretion in HEK293 cells. (a) DN13‐DN1 nanobody binding to the eight human Snap‐tagged mGlu receptor subtypes transiently expressed in HEK293T cells and co‐transfected with the high‐affinity glutamate transporter EAAC1. Binding was measured in the presence of saturating concentrations of subtype‐specific agonists (1 µM quisqualate for mGluR1 and mGluR5; 10 µM L‐AP4 for mGluR4, 6, 7, and 8; 1 µM LY379268 for mGluR2 and mGluR3), the antagonist LY341495 (10 µM), or buffer. TR‐FRET signal was detected using Snap‐Lumi4‐Tb as the donor and an anti‐6His antibody conjugated to the d2 acceptor fluorophore. Data represent mean ± s.e.m. from three biologically independent experiments, each performed in triplicate. (b) Potentiation of IP_1_ accumulation by DN13‐DN1 and DN1 nanobodies in HEK293T cells transiently co‐expressing mGluR2, EAAC1, and the chimeric G protein Gqi9, enabling mGluR2 coupling to the phospholipase C pathway. Cells were stimulated in the presence of an EC_20_ concentration of LY379268 (0.88 nM). Data are shown as mean ± s.e.m. from three biologically independent experiments, each performed in triplicate. (c) Schematic representation of the TR‐FRET‐based assay used to quantify Aβ_1‐42_ levels in HEK293T cells expressing endogenous human APP (eAPP), or transfected with a human Halo‐tagged APP (^HT^APP) and transfected with Snap‐tagged mGluR2 or mGluR3 constructs (^ST^mGluR2 or ^ST^mGluR3). Created with BioRender.com. (d–g) Levels of secreted Aβ_1‐42_ produced from eAPP (*d*) or ^HT^APP (*e*) and secreted levels of sAPPβ (*f*) and sAPPα (*g*) in HEK293T cells transfected with either ^ST^mGluR2 or ^ST^mGluR3, and co‐transfected or not with ^HT^APP. Cells were treated with PBS, LY379268 (5 µM) alone, LY379268 (5 µM) following pre‐treatment with the antagonist LY341495 (10 µM), DN13‐DN1 (200 nM) alone, LY379268 at EC_20_ (0.5 nM) alone, or the combination of DN13‐DN1 (200 nM) with LY379268 at EC_20_. Controls included cells transfected with an empty vector (Mock) or ^HT^APP alone. Data are presented as mean ± s.e.m. from three biologically independent experiments, each performed in triplicate, and are presented as a ratio of the basal levels. Data were analyzed using the one‐way ANOVA followed by a Holm‐Sidak's *post‐hoc* analysis (* *p* < 0.05, ** *p* < 0.01, *** *p* < 0.001).

To investigate the specific roles of mGluR2 and mGluR3 in Aβ_1‐42_ production and secretion, we examined the effects of their activation on APP processing in HEK293 cells. To activate mGluR2, we used DN13‐DN1, a recently developed biparatopic nanobody [22], that we compared to the non‐selective mGluR2 and mGluR3 agonist LY379268. This nanobody has a high affinity for both mouse and human mGluR2 orthologs on HEK293 cells (pKd = 9.24 ± 0.01 and 9.69 ± 0.18, respectively) [22] and specifically recognizes mGluR2 among all other mGluRs (Figure 1a, Figure S1a,b). DN13‐DN1 exhibits a positive allosteric modulatory (PAM) effect with a high potency on both mouse (pEC_50_ = 8.77 ± 0.42) and human (pEC_50_ = 8.62 ± 0.03) orthologs [22] (Figure 1b, Figure S1c).

Production of Aβ_1‐42_ peptides in HEK293T cells upon activation of mGluR2 or mGluR3 was measured by using a time‐resolved fluorescence energy transfer (TR‐FRET)‐based assay (Figure 1c). Cells expressing an endogenous human APP (eAPP) were transfected with a Snap‐tagged mGluR2 or mGluR3 construct [28], called ^ST^mGluR2 and ^ST^mGluR3, respectively. Activation of ^ST^mGluR2 increased significantly the production and secretion of Aβ_1‐42_, while activation of ^ST^mGluR3 did not (Figure 1d). This secretion is stimulated when ^ST^mGluR2 is activated with a saturating concentration of DN13‐DN1 nanobody alone, or in combination with the orthosteric full agonist LY379268 at its EC_20_. This production of Aβ_1‐42_ is similar to the condition using a saturating concentration of LY379268. It is also blocked by a saturating concentration of the non‐selective mGluR antagonist LY341495 (Figure 1d). Of note, the increase of Aβ_1‐42_ secretion induced by DN13‐DN1 alone might be due to the presence of ambient glutamate in the medium. In addition, we show that the absence of production of Aβ_1‐42_ peptides in ^ST^mGluR3 transfected cells is not due to a lower cell surface expression of the receptor compared to ^ST^mGluR2, using the non‐cell permeant luminescent terbium cryptate donor (Lumi4‐Tb) to label the Snap‐tag receptors (Figure S2a), including in conditions where the receptors were treated with the nanobody or with agonist (Figure S2b).

Similar results were obtained when the human Halo‐tagged APP (^HT^APP) was overexpressed in HEK293T cells co‐transfected with ^ST^mGluR2 or ^ST^mGluR3 in which activation of mGluR2 promotes Aβ_1‐42_ peptides production, but not mGluR3 (Figure 1e). Interestingly, pre‐incubation with LY341495, or with the pertussis toxin (PTX), known to inhibit G_i/o_ signaling, (Figure S3a), strongly reduced the secreted levels of Aβ_1‐42_ upon activation of mGluR2, indicating a process dependent of the G protein activation by the receptor. We verified that the absence of production of Aβ_1‐42_ peptides in co‐transfected ^HT^APP and ^ST^mGluR3 cells was not due to the lower cell surface expression of these constructs, using either the non‐cell permeant Tag‐lite Snap or Halo‐Lumi4‐Tb labeling reagents (Figure S2c–e), including in conditions where the receptors were treated with the nanobody or with agonist (Figure S2d).

To investigate whether mGluR3 could have any role in APP processing, we examined the effects of its activation on the production and secretion of the soluble APPβ fragments (sAPPβ, generated after β‐secretase cleavage) and sAPPα (generated after α‐secretase cleavage) assayed by ELISA (Figure 1c) in HEK293T cells. Cells were co‐transfected with ^HT^APP and with ^ST^mGluR2 or ^ST^mGluR3 and were treated in the same conditions as explained above. As expected, activation of ^ST^mGluR2 increased significantly the production and secretion of sAPPβ, while activation of ^ST^mGluR3 did not (Figure 1f, Figure S3b). In contrast, activation of ^ST^mGluR3 rather stimulated the production and secretion of sAPPα fragments (Figure 1g), and pre‐incubation with LY341495, or with the PTX, strongly reduced the secreted levels of sAPPα after activation of ^ST^mGluR3 (Figure S3c). This shows a process dependent of the G protein activation by the receptor. We also verified that it was not due to the lower cell surface expression of these constructs (Figure S2f–h).

Taken together these data indicate that activation of mGluR2 contributes to the production and secretion of Aβ_1‐42_ peptides, at least in part, through a G protein‐dependent mechanism. In contrast, activation of mGluR3 does not induce amyloid peptide production but rather shifts APP processing towards the non‐amyloidogenic pathway, in a G protein‐dependent manner.

### 2.2 mGluR3 Directly Interacts With APP, but not mGluR2

**FIGURE 2:**
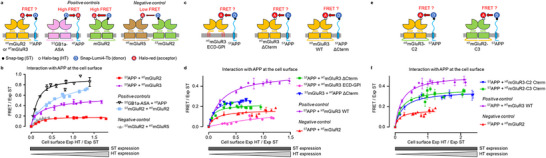
APP interacts with mGluR3, but not mGluR2, via its transmembrane and C‐terminal domains. (a) Schematic of the constructs used to assess APP interaction with mGluR2 or mGluR3. (b) TR‐FRET saturation analyses in HEK293T cells co‐expressing Snap‐tagged APP (^ST^APP) with Halo‐tagged mGluR3 (^HT^mGluR3; purple) or ^HT^mGluR2 (red); ^ST^mGluR2 with ^HT^mGluR2 (blue, positive control) or with ^HT^mGluR5 (grey, negative control); and ^ST^GB1a‐ASA, a construct known to reach constitutively the cell surface, with ^HT^APP (black, positive control). Snap‐tagged constructs were transfected at a fixed cDNA amount and labelled with the donor (Snap‐Lumi4‐Tb labeling reagent), while increasing amounts of Halo‐tagged receptors were labelled with the acceptor (Halo‐red labeling reagent). Expression of cell surface Snap‐tagged (Exp ST) and Halo‐tagged (Exp HT) receptors as well as the FRET signal were measured. FRET/Exp ST is plotted as a function of the Halo to Snap expression ratio (Exp HT/Exp ST). Data are presented as mean ± s.e.m. of triplicates from a representative experiment, repeated three times. (c) Schematic of the constructs used to identify the domains of mGluR3 mediating its interaction with APP. (d) TR‐FRET saturation analyses in HEK293T cells co‐expressing ^ST^APP with either ^HT^mGluR3 ECD‐GPI (Del570‐879; pink), a construct where the transmembrane and C‐terminal regions were replaced with a GPI anchor; a C‐terminally truncated variant ^HT^mGluR3‐ΔCterm (Del829‐879; green), ^HT^mGluR2 (red, negative control), or with ^HT^mGluR3 (purple, positive control). Additional TR‐FRET experiments were conducted with ^ST^mGluR3 co‐expressed with a C‐terminally truncated ^HT^APP (^HT^APP‐ΔCterm, Del648–695; blue). Data are presented as mean ± s.e.m. of triplicates from a representative experiment, repeated three times. (e) Schematic of the constructs to assess APP interaction with mGluR2 and mGluR3 chimeras in which the C‐terminal domains were swapped. (f) TR‐FRET saturation analyses in HEK293T cells co‐expressing ^ST^APP with ^HT^mGluR3 chimera (^HT^mGluR3‐C2; blue), ^HT^mGluR2 chimera (^HT^mGluR2‐C3; green), ^HT^mGluR2 (red, negative control), or ^HT^mGluR3 (purple, positive control). Data are presented as mean ± s.e.m. of triplicates from a representative experiment, performed three times.

We next investigated why mGluR2 and mGluR3 differ in their effects on Aβ_1‐42_ production, despite sharing approximately 81% amino acid sequence similarity [16] and common signaling pathways through G_i/o_ proteins. We hypothesized that this divergence could result from differences in their ability to interact with APP, an aspect that had not yet been explored (Figure 2a). To assess potential interaction between APP and either mGluR2 or mGluR3 at the cell surface of living cells, we performed TR‐FRET saturation assays using orthogonal labeling of the two protein partners in HEK293T cells [29] (Figure 2b, Figure S4). In this assay, the FRET signal is measured between the Snap‐ and Halo‐tagged constructs labelled with the non‐cell‐permeant Tag‐lite Snap‐Lumi4‐Tb and Halo‐Red labeling reagents, in which a fixed amount of the Snap‐tagged construct is co‐transfected with increasing amounts of the Halo‐tagged construct. As a positive control, we co‐transfected a Snap‐tagged GB1a construct (^ST^GB1a‐ASA) known to reach constitutively the cell surface and produced a FRET saturation signal with ^HT^APP (Figure 2b, Figure S4). Indeed, it has been described that APP can bind directly to the N‐terminal sushi domain of the GABA_B1a_ (GB1a) subunit of the GABA_B_ receptor, also belonging to the class C GPCRs like the mGlu receptors, and this interaction limits the production of the Aβ peptides in the endosomal compartment [30]. By contrast, as a negative control, the co‐expression of ^ST^mGluR2 with ^HT^mGluR5, two mGlu protomers that are unable to form heterodimers, exhibited a low FRET signal, as expected [29]. Co‐expression of ^ST^APP and ^HT^mGluR3 exhibited a high FRET saturation curve, reflecting a direct interaction of APP with mGluR3 (Figure 2b, Figure S4). This shows that mGluR3 has a strong tendency to form a complex with APP. In contrast, co‐expression of the ^ST^APP and ^HT^mGluR2 constructs exhibited a low FRET saturation curve, comparable to the one observed for cells expressing ^ST^mGluR2 with ^HT^mGluR5, indicating that mGluR2 and APP do not interact. This conclusion is further supported by a recent interactome analysis of the mouse prefrontal cortex using the DN13‐DN1 nanobody, which did not identify APP among the significant interacting proteins [31].

To characterize the molecular basis of the interaction between APP with mGluR3, we performed TR‐FRET saturation assays by using APP and mGluR3 mutants. The FRET saturation is suppressed in cells co‐expressing ^ST^APP and the isolated extracellular domain (ECD) of mGluR3, in which the transmembrane and C‐terminal regions were replaced with a GPI anchor (^HT^mGluR3 ECD‐GPI, Figure 2c,d, Figure S5a,b). This suggests that the transmembrane and intracellular C‐terminal regions of mGluR3 are critical for the interaction with APP. To validate the implication of the intracellular domain of mGluR3 in this interaction, a construct with a deletion of the C‐terminal domain of ^HT^mGluR3 (ΔCterm) was used, revealing a strong decrease in FRET saturation (Figures 2c‐d, Figure S5a,b). Reciprocally, deletion of the intracellular C‐terminal region of APP (ΔCterm) also showed a strong reduction of FRET saturation with ^ST^mGluR3 (Figure 2c,d, Figure S5a,b). We next exchanged the C‐terminal regions of mGluR2 and mGluR3 (^ST^mGluR2‐C3 and ^ST^mGluR3‐C2, respectively) (Figure 2e). Interestingly, we observed that ^ST^mGluR2‐C3 co‐transfected with ^HT^APP, exhibited an increased FRET signal compared to ^ST^mGluR2 (Figure 2f, Figure S5c,d). However, a decrease in the FRET saturation signal was observed with the ^ST^mGluR3‐C2 construct compared to ^ST^mGluR3.

To confirm in native tissues that mGluR3 directly interacts with APP, but not mGluR2, we performed immunoprecipitations of the endogenous receptors from mouse cortex tissues, using specific nanobodies. We used the DN13‐DN1 nanobody for mGluR2 [22] and a silent allosteric ligand mGluR3‐specific nanobody with nanomolar affinity. APP co‐immunoprecipitated with the endogenous mGluR3 (Figure S6a), but not with mGluR2 (Figure S6b), in western blotting analysis. In the negative control, no APP was detected when the cortex extracts were incubated with agarose beads without any nanobody (Figure S6).

Altogether, these data indicate that mGluR3 specifically associates with APP, unlike mGluR2, and that this interaction is mediated by both the transmembrane and C‐terminal regions of the receptor.

### 2.3 APP Is Internalized Upon mGluR2 Activation

**FIGURE 3:**
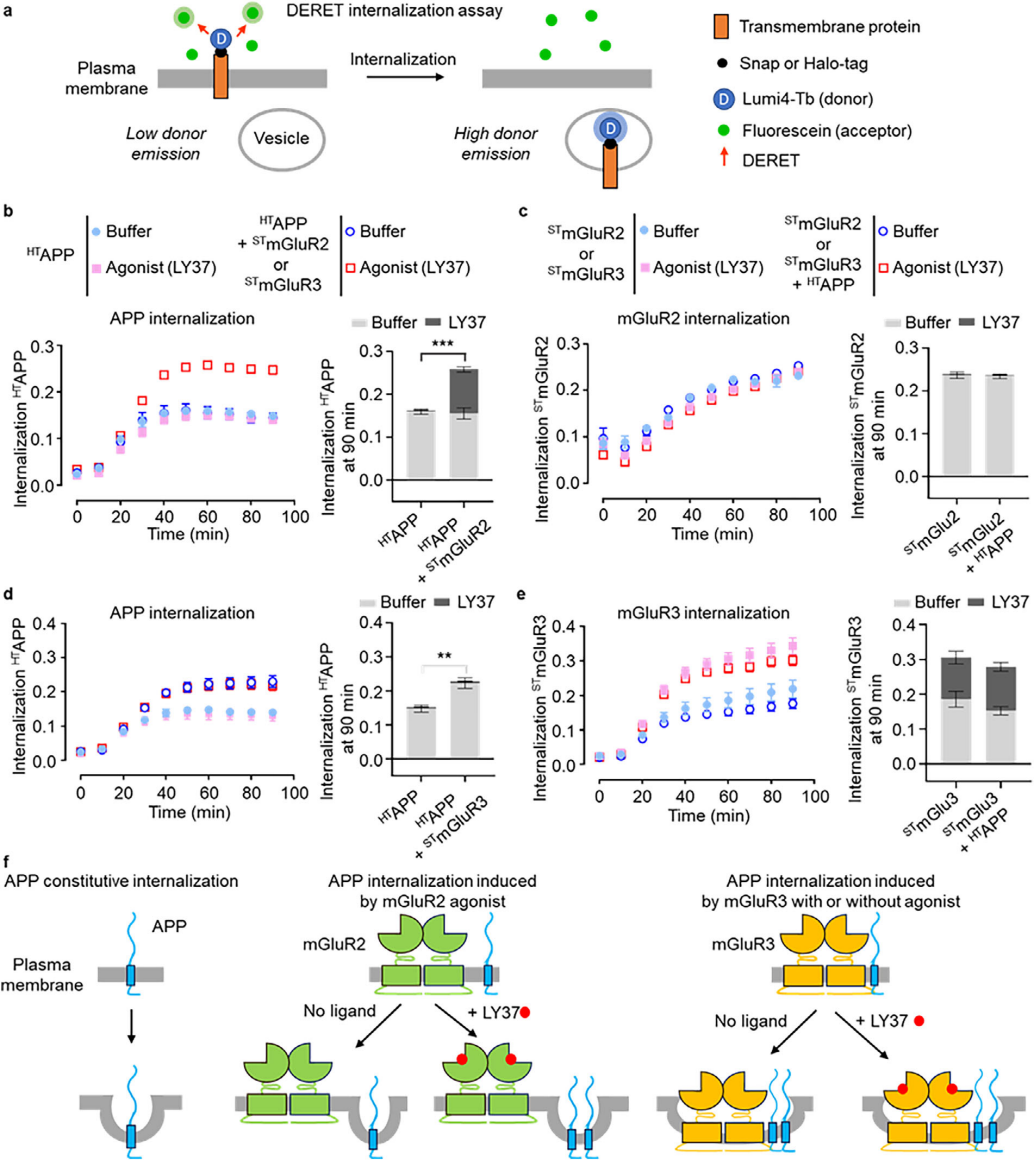
APP is internalized upon mGluR2 activation. (a) Schematic representation of the DERET (diffusion‐enhanced resonance energy transfer)‐based internalization assay. Created with BioRender.com. (b,c) Kinetics of internalization of Halo‐tagged APP (^HT^APP, (b)) and Snap‐tagged mGluR2 (^ST^mGluR2, (c)) in HEK293T cells, transfected individually or together. Cells were treated with buffer (PBS) or with 1 µM of LY379268 (LY37). Bars represent constitutive internalization (light grey, basal signal) and agonist‐induced internalization (dark grey, calculated by subtracting basal signal), measured at 90 min of internalization. (d,e) Internalization of ^HT^APP (*d*) and Snap‐tagged mGluR3 (^ST^mGluR3, *e*) under the same experimental conditions as in (b,c). Data are presented as mean ± s.e.m. from three biologically independent experiments, performed in triplicates. Statistical analysis was performed using Welch's t test (** *p* < 0.01, *** *p* < 0.001). (f) Proposed model of APP internalization dynamics. APP undergoes constitutive internalization. Upon mGluR2 activation by LY379268, APP internalization is increased. In the presence of mGluR3, with or without ligand, APP internalization is increased.

^HT^APP, when expressed alone, exhibits a constitutive internalization (Figure 3b), consistent with its known intracellular trafficking [32, 33], which is not altered when co‐expressed with ^ST^mGluR2. However, the activation of ^ST^mGluR2 with LY379268 enhanced ^HT^APP internalization, showing that mGluR2 activation promotes APP endocytosis. ^ST^mGluR2 also displayed constitutive internalization (Figure 3c), as previously reported [34], and co‐transfection with ^HT^APP did not impact its trafficking either in buffer or agonist conditions. We verified that the cell surface expression of ^ST^mGluR2 and ^HT^APP, alone or co‐transfected, were similar (Figure S7a). In addition, we tested whether mGluR2 agonist‐induced APP endocytosis implicates β‐arrestins, as shown for another GPCR [36, 37]. We show that ^HT^APP constitutive internalization (Figure S8a,b) and mGluR2 agonist‐induced ^HT^APP internalization (Figure S8b) in β‐arrestin knockout HEK293 cells [38] transfected with ^HT^APP and ^ST^mGluR2, alone or together, were unaffected in our DERET internalization assay. In contrast, mGluR2 constitutive internalization, when ^ST^mGluR2 is transfected alone or co‐transfected with APP, was strongly reduced in these β‐arrestin knockout cells (Figure S8c,d), consistent with established mGluR2 trafficking mechanisms [34]. Altogether, these data demonstrate that agonist mGluR2‐induced APP endocytosis is β‐arrestin‐independent.

To understand the cellular mechanisms enhancing Aβ_1‐42_ production following mGluR2 activation, we analyzed the kinetics of APP internalization, either its constitutive or mGluR‐induced internalization. This step is critical, as APP must be internalized into the endosomal compartment to undergo amyloidogenic cleavage by the β‐secretase [32, 33]. To this end, we assessed the internalization kinetics using diffusion‐enhanced resonance energy transfer (DERET) [34, 35], in HEK293T cells co‐transfected with ^HT^APP and ^ST^mGluR2 or ^ST^mGluR3. The fluorescence of ^HT^APP or ^ST^mGluRs labelled with a non‐cell permeable fluorophore (energy donor), which is quenched with an excess of free fluorescent quencher in the medium (energy acceptor), is recovered upon their internalization (Figure 3a).

In contrast, ^HT^APP constitutive internalization is increased when co‐expressed with ^ST^mGluR3 (Figure 3d). This increase reaches the same level as the constitutive internalization of ^ST^mGluR3 (Figure 3e) indicating that APP is co‐internalized with mGluR3. Interestingly, agonist stimulation of ^ST^mGluR3 did not further increase APP internalization (Figure 3d). We verified that ^ST^mGluR3 expressed alone exhibited a constitutive internalization and was further internalized upon LY379268 agonist treatment (Figure 3e), as previously reported [34, 39], and both were not modified when co‐expressed with ^HT^APP. Finally, we checked that the cell surface expression of ^ST^mGluR3 and ^HT^APP, alone or co‐transfected, were similar (Figure S7b).

As the mGluR3‐APP complex increases APP internalization without inducing the production of Aβ peptides, we investigated the intracellular trafficking of this complex using different endosomal markers. To this end, ^HT^APP and ^ST^mGluR3 were co‐transfected and labeled with the non‐cell permeant fluorophores Halo‐Alexa Fluor 488 (green) and Snap‐Alexa Fluor 594 (red), respectively. The early endosomal and the recycling endosomal were identified using the markers Rab5 and Rab11, respectively (Figure S9). APP transfected alone colocalized with Rab5^+^ and Rab11^+^ endosomal markers, suggesting that APP internalized constitutively is mainly recycled to the plasma membrane (Figure S9a,d,e). When co‐transfected with ^ST^mGluR3, colocalization analyses of ^HT^APP with either Rab5 or Rab11 markers confirmed that the APP‐mGluR3 complex internalizes into Rab5^+^ early endosomes (Figure S9b–d), in both basal and agonist conditions. Interestingly, the same proportion of ^HT^APP found in early endosomes, also colocalizes in the Rab11^+^ recycling endosomes (Figure S9b,c,e), suggesting that most APP co‐internalized with mGluR3 is recycled to the plasma membrane. In addition, upon LY379268 mGluR3 agonist treatment, the colocalization of ^HT^APP and ^ST^mGluR3 is increased (Figure S9f), which may due to the agonist‐induced internalization of mGluR3. In addition, as observed in our DERET experiments (Figure 3d), ^HT^APP internalization increases when ^ST^mGluR3 is co‐transfected (Figure S9d,e). This increase may reflect that APP internalization reaches a level comparable to the constitutive internalization of ^ST^mGluR3.

Altogether, our results show that APP internalization is modulated by both mGluR2 and mGluR3 (Figure 3f). Activation of mGluR2 increases APP internalization, potentially promoting its targeting to endosomal compartments where amyloidogenic processing into Aβ peptides occurs. This is consistent with our observation that mGluR2 activation enhances Aβ_1‐42_ and sAPPβ production (Figure 1d–f). In contrast, APP constitutive internalization is increased in the presence of mGluR3, consistent with their ability to interact directly (Figure 2b), but notably this internalization is not further accentuated upon mGluR3 activation (Figure 3f). The co‐internalization of APP‐mGluR3 complex directs APP trafficking toward recycling endosomes (Figure S9g), which likely explains the lack of Aβ_1‐42_ peptide production (Figure 1d,e) following this internalization.

### 2.4 Chronic Administration of DN13‐DN1 Nanobody Aggravates Cognitive Deficits in 5xFAD Mice

**FIGURE 4:**
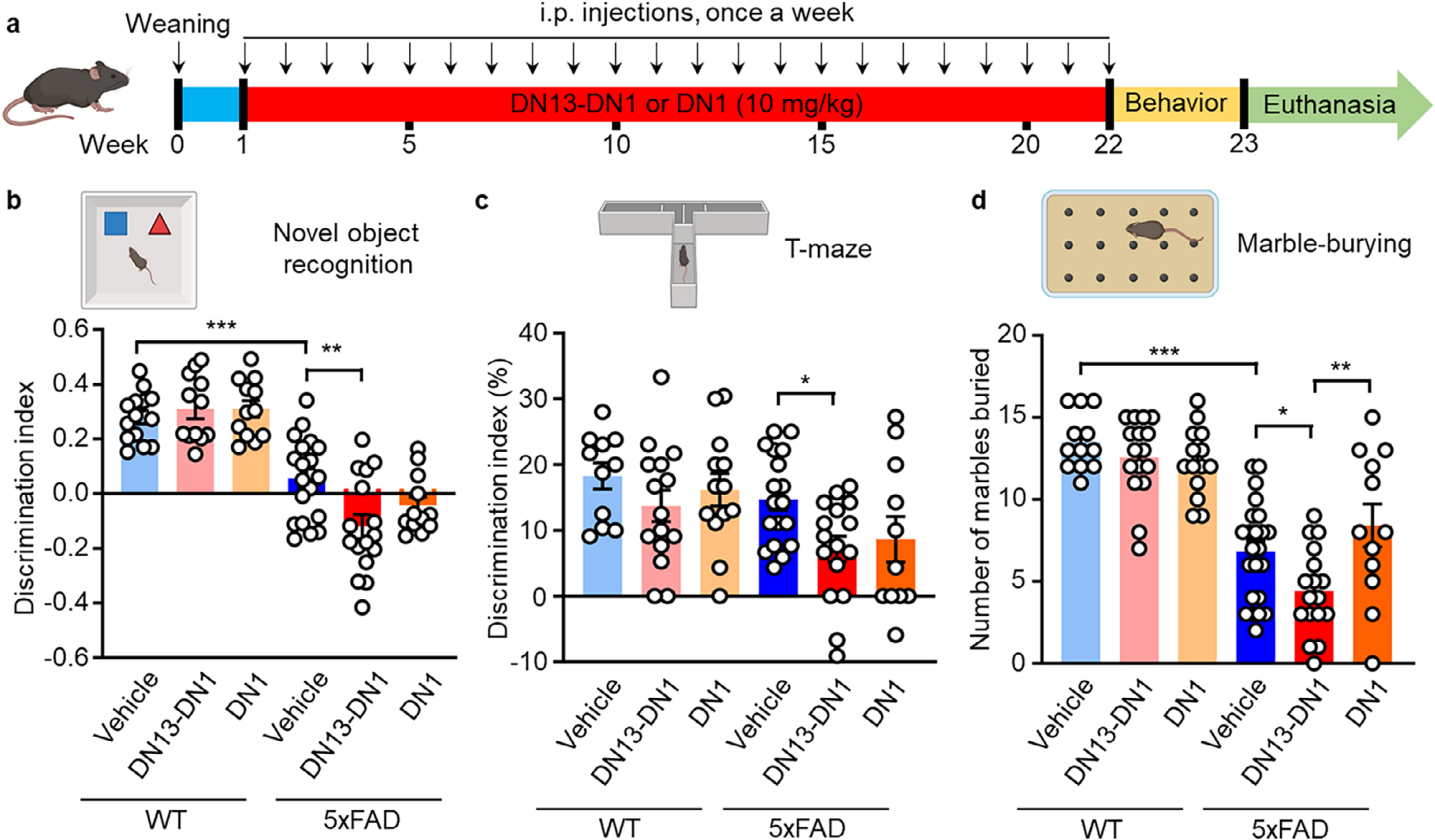
Positive modulation of mGluR2 by DN13‐DN1 nanobody worsens cognitive deficits in 5xFAD mice. (a) Schematic representation of the chronic administration of nanobodies in wild‐type (WT) and 5xFAD mice. One week post‐weaning (4 weeks old), male 5xFAD and WT littermates were treated intraperitoneally with 10 mg/kg of the DN13‐DN1 nanobody, the mGluR2 silent allosteric ligand DN1 nanobody, or with an equivalent volume of vehicle. Injections were administered once a week for 22 weeks. Following treatment, mice underwent several behavioral tests, including nest‐building, marble‐burying, T‐maze spontaneous alternation, and novel object recognition. (b–d) Behavioral performance of WT and 5xFAD mice across treatment groups. (b) Number of marbles buried in the marble burying test. (c) Discrimination index in the T‐maze spontaneous alternation. (d) Discrimination index in the novel object recognition test. Data are presented as mean ± s.e.m. (*n* = 12‐21 mice/group) and were analyzed using the two‐way ANOVA followed by a Holm‐Šidák *post hoc* analysis (* *p* < 0.05, ** *p* < 0.01, *** *p* < 0.001). Created with BioRender.com.

To validate the amyloidogenic potential of mGluR2 in vivo, we used the 5xFAD transgenic mouse model which recapitulates cognitive and behavioral impairments driven by Aβ_1‐42_ overproduction [40]. These mice were chronically treated with the DN13‐DN1 nanobody, which acts as a positive allosteric modulator of mGluR2 (Figure 4a). This nanobody reaches the brain following intraperitoneal injections and remains detectable for up to 7 days post‐administration [22]. Mice were treated at 4 weeks of age, once a week, for 22 weeks, through intraperitoneal injections (10 mg/kg) until the age of 6.5 months, where cognitive deficits in this mouse model are measurable [40].

Using the novel object recognition test (NOR), assessing the long‐term memory, we first demonstrate that 5xFAD mice displayed decreased exploration of the novel object as shown by a decreased discrimination index (Figure 4b). This deficit was further aggravated by the DN13‐DN1 chronic treatment (10 mg/kg, weekly). In contrast, chronic treatment at the same dose with the DN1, a neutral nanobody specific to mGluR2 without any pharmacological properties [22, 24], is not significantly different from the 5xFAD vehicle mice, demonstrating that the PAM property of DN13‐DN1 is crucial for observing behavioral changes. WT littermates were unaffected by the same treatments.

The transgenic 5xFAD mouse model can also display impairments in spatial working memory that can be assessed using the T‐maze test [41]. DN13‐DN1 treatment significantly reduced the discrimination index (Figure 4c), suggesting worsened spatial and short‐term memory loss. We confirmed that DN1 treatment did not modify significantly the discrimination index of 5xFAD mice and that WT mice were unaffected by both treatments. In addition, anxiety, assessed by the marble‐burying test, is also affected in 5xFAD mice that bury less marbles (Figure 4d), reflecting a less anxious behavior. This phenotype is exacerbated when 5xFAD was treated with DN13‐DN1, but not with the DN1 nanobody. To note, treatment of WT mice with the nanobodies did not modify the burying behavior. No effect of nanobodies was observed in other behavioral tests not related to cognition. Indeed, neither DN13‐DN1 nor DN1 modified the ability of animals to build nests, a natural behavior of mice that requires hippocampal neuronal functions and reports on hippocampal integrity (Figure S10a,b). In addition, no effect of the chronic treatment was also observed in locomotor parameters measured in an open field, such as time spent in center, velocity, distance, and immobility (Figure S10c–f).

In addition, chronic treatment with both nanobodies did not modify the weight of animals in any group (Figure S11a) and had minimal effect on mice survival (Figure S11b). In addition, no apparent toxicity was observed in a histopathological study performed on 27 organs collected in the WT mice chronically treated with DN13‐DN1 and DN1 (Table S1). We only observed in the kidneys some tubular basophilia that may be related to alterations in the functional activity of the tubular cells rather than toxicity.

Altogether, these data demonstrate that chronic modulation of mGluR2 using the DN13‐DN1 nanobody worsens both spatial and recognition memory deficits in 5xFAD mice, while no behavioral effects were observed in WT control mice.

### 2.5 DN13‐DN1 Nanobody Increases Amyloid Load and Inflammatory Processes in 5xFAD Mice

**FIGURE 5:**
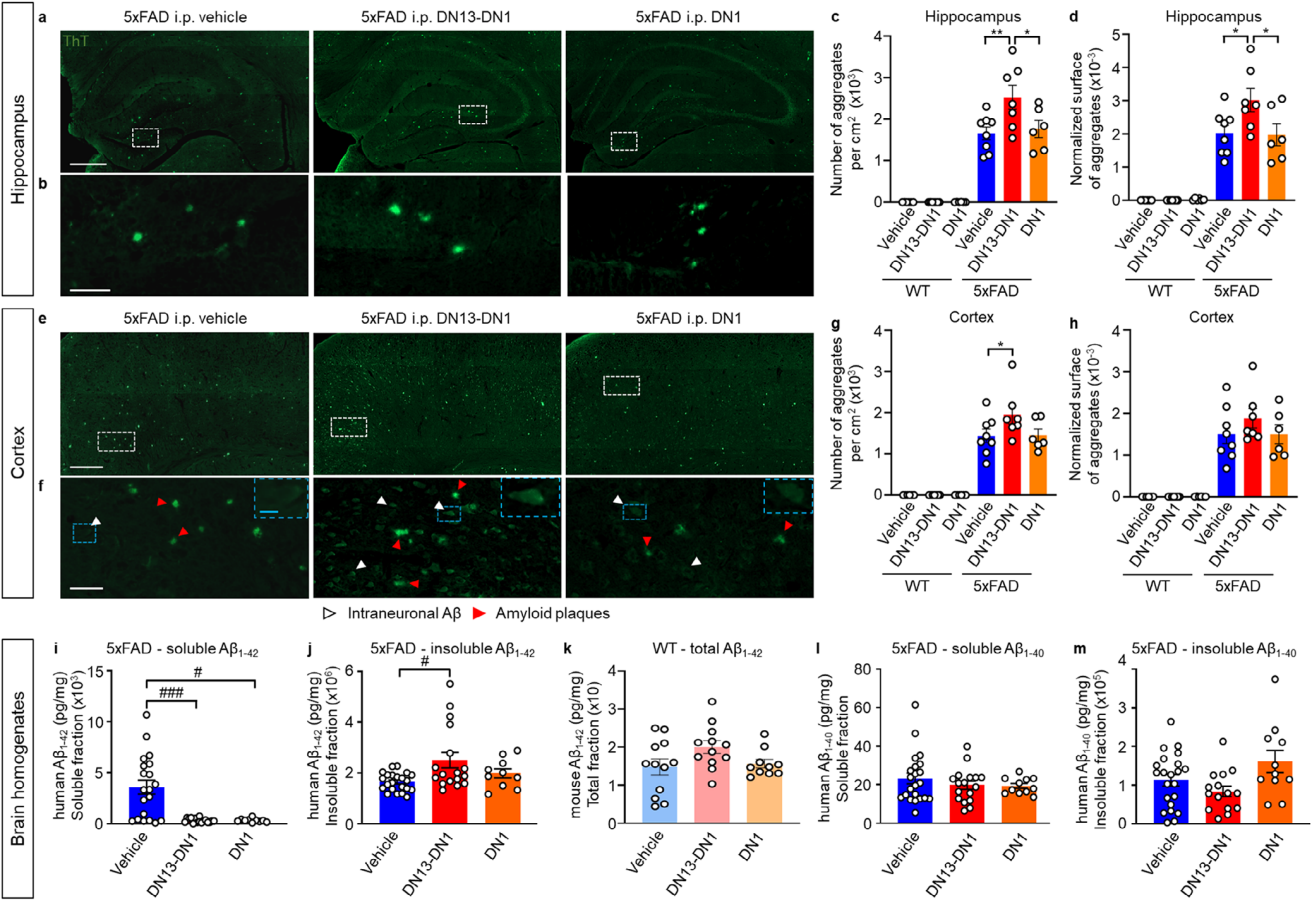
DN13‐DN1 nanobody treatment worsens amyloid pathology in 5xFAD mice. (a,b) Representative images of thioflavin T (ThT) staining showing amyloid plaques in the hippocampus of 26‐week‐old 5xFAD mice chronically treated with 10 mg/kg of either DN13‐DN1, DN1, or with vehicle. Whole‐section mosaic images were acquired using a slide scanner Axio Scan.Z1 with a 20x objective (a, scale bar: 200 µm); high‐magnification views were obtained via digital zoom (b, scale bar: 50 µm). (c,d) Quantification of plaque number (c) and surface area (d) normalized by the total hippocampus area analyzed in WT and 5xFAD mice. (e,f) Representative ThT‐stained amyloid plaques in the cortex of 5xFAD mice in some the same treatment groups. Images were acquired as mosaics using a 20× objective (e, scale bar: 200 µm); enlarged views were generated by digital zoom (f, scale bar: 50 µm). Insets in blue show individual neurons at higher magnification (f, scale bar: 10 µm). White and red arrowheads highlight intraneuronal Aβ and amyloid plaques, respectively. (g,h) Quantification of the plaque number (g) and surface (h) normalized by the total cortical area in WT and 5xFAD mice. Data in panels (c,d) and (g,h) are presented as mean ± s.e.m. (*n* = 6–8 animals/group, 13–23 sections/animal) and analyzed using the two‐way ANOVA followed by a Holm‐Šidák *post hoc* analysis (* *p* < 0.05; ** *p* < 0.01). (i–m) Biochemical quantification of amyloid species in hemibrains. Soluble (i) and insoluble (j) fractions of human Aβ_1‐42_ in 5xFAD hemibrains measured by a HTRF‐based assay. (k) Endogenous mouse Aβ_1‐42_ levels in WT hemibrains quantified by ELISA. (l,m) Soluble (l) and insoluble (m) fractions of human Aβ_1‐40_ in 5xFAD hemibrains measured by FRET‐based assay. Data are presented as mean ± s.e.m. (*n* = 10–22 animals/group). Statistical analyses were performed using Kruskal‐Wallis followed by a Dunn's multiple comparisons for (i–l) (# *p* < 0.05, ## *p* < 0.01) and one‐way ANOVA followed by a Holm‐Šidák *post hoc* test for *m*.

Given that DN13‐DN1 exacerbates cognitive deficits in 5xFAD mice, we next assessed the impact of the chronic nanobody treatment on amyloid load in both WT and 5xFAD animals (Figure 5). We first quantified amyloid aggregates using the Thioflavin T (ThT) staining on hemibrain sections (Figure S12). We showed that DN13‐DN1 treatment in 5xFAD strongly increased both the number of amyloid plaques (+ 52%) (Figures 5a‐c) and the surface of the aggregates (+ 49%) (Figures 5a‐b, d) in the hippocampus. Similarly, in the cortex, a significant increase of the number of aggregates (+36%) (Figure 5e–g), but not of their surface (Figure 5e–f,h), were quantified. In agreement with the absence of effects on behavioral tests with the DN1, no impact on the amyloid aggregates was measured with this nanobody for both hippocampus and cortex (Figure 5). WT littermates were unaffected by DN1 and DN13‐DN1 treatments, with no aggregates detectable. We then examined the presence of intraneuronal Aβ accumulation, one of the hallmarks of the 5xFAD mouse model [40]. We showed that treatment with the DN13‐DN1 nanobody increased the proportion of intraneuronal ThT fluorescence in cortical neurons (Figure 5f, inset), an effect also measured with DN1 treatment, but in a lesser extent.

To further investigate the molecular basis of these histological observations, we conducted quantitative analyses of Aβ_1‐42_ and Aβ_1‐40_ levels in brain homogenates of 5xFAD and WT mice using a homogeneous time‐revolved fluorescence (HTRF^TM^) assay [42, 43] or ELISA [44]. Surprisingly, 5xFAD mice chronically treated with DN13‐DN1 and DN1 nanobodies exhibited a strong reduction of Aβ_1‐42_ soluble fraction (Figure 5i). Consistent with the increased amyloid plaque load, quantification showed a clear increase of the insoluble Aβ_1‐42_ fraction in brain samples from 5xFAD mice treated with the DN13‐DN1 nanobody (+52%) (Figure 5j). This suggests that the chronic administration of DN13‐DN1 nanobody promotes the aggregation of soluble Aβ_1‐42_ into insoluble amyloid plaques, reflecting a shift in Aβ_1‐42_ distribution. In contrast, DN1 nanobody treatment did not modify the Aβ_1‐42_ insoluble levels (Figure 5j). Interestingly, chronic treatment of WT mice with the nanobodies did not modify the total mouse Aβ_1‐42_ levels compared to the WT vehicle‐treated mice (Figure 5k). This finding reveals that the DN13‐DN1 nanobody specifically affects Aβ_1‐42_ production only in the pathological context of the 5xFAD model but not in the physiological condition. Unlike Aβ_1‐42_ peptides, no differences were observed for Aβ_1‐40_ levels on total brain homogenates upon chronic administration of DN13‐DN1 or DN1 nanobodies for both soluble (Figure 5l) and insoluble (Figure 5m) fractions.

As the DN13‐DN1 chronic treatment increases the amyloid burden in 5xFAD mice, we next assessed its impact on inflammatory processes. We first quantified astrogliosis using the GFAP marker by immunofluorescence on hemibrain sections (Figure S13). We showed that DN13‐DN1 treatment in 5xFAD significantly increased the intensity of the labelling (Figure S13a,b) but not the surface of the labelling (Figure S13a,c) in the cortex. In contrast, in the hippocampus, no significant increase of the intensity (Figure S13d–e) and the surface of the labelling (Figure S13d, f) were quantified. As expected, no increase of astrogliosis was quantified in DN1‐treated 5xFAD mice in the cortex or hippocampus (Figure S13). WT littermates were unaffected by both treatments, with no increased astrogliosis. Regarding microgliosis, analyzed using the IBA1 marker (Figure S14), DN13‐DN1 treatment increases both intensity (Figure S14a,b) and surface of the labelling (Figure S14a, c) in the cortex, but only in the intensity of labelling in the hippocampus (Figure S14d–f).

Altogether, these data show that chronic administration of the DN13‐DN1 nanobody exacerbates amyloid pathology in the 5xFAD model by increasing Aβ_1‐42_ production and enhancing the aggregation process, without altering overall Aβ_1‐42_ levels in healthy brains. This increased amyloid burden is associated to exacerbated inflammatory processes. Altogether, these findings highlight the context‐dependent effects of DN13‐DN1 in modulating amyloid production.

### 2.6 DN13‐DN1 Decreases mGluR2 Expression in the Cortex of 5xFAD Mice

DN13‐DN1 treatment had a lower impact on the amyloid load in the cortex of 5xFAD mice compared to the hippocampus (Figure 5). We hypothesized that region‐specific changes in mGluR2 expression might underline this discrepancy. Indeed, mGluR2 expression can be modulated by treatments including antipsychotics, through epigenetic mechanisms [45, 46]. Furthermore, mGluR2 agonists have been reported to modulate receptor signaling while also affecting receptor regulation and desensitization, as it is typical of GPCRs [47]. To explore whether chronic DN13‐DN1 treatment alters mGluR2 expression in a region‐specific manner, we quantified mGluR2 levels using a nanobody‐based FRET assay. This approach used DN1 nanobody coupled to a donor (DN1‐Tb) and an acceptor (DN1‐d2) fluorophore (Figure S15a), as previously described [22, 26], on dissociated cells from various brain regions. In the cortex, we showed a significant reduction in mGluR2 expression in vehicle‐treated 5xFAD mice compared to their WT littermates (Figure S15b), an effect that was further exacerbated following the chronic treatment with DN13‐DN1. No alterations in mGluR2 expression were detected in the hippocampus (Figure S15c) or the cerebellum (Figure S15d) between control and treated 5xFAD and WT mice. To note, chronic treatment with either DN13‐DN1 or DN1 nanobodies did not affect the expression of mGluR2 in WT mice (Figure S15a–d). As a negative control, we used the midbrain as mGluR2 is not expressed in this region [26] (Figure S15a).

To explore whether this reduction of mGluR2 expression in the cortex could reflect increased neuronal cell death in this region, associated with mGluR2‐dependent Aβ_1‐42_ production, we quantified the neurons in the cortex on hemibrain sections using cresyl violet staining (Figure S16). We observed a genotype reduction in the total number of neurons in the cortex (Figure S16a,b) associated with a decreased number of viable neurons (Figure S14c) and an increased number of damaged neurons (Figure S16d). However, chronic treatment of 5xFAD mice with the DN13‐DN1 does not accentuate this neuronal loss (Figure S16a–d).

Altogether, these findings show that chronic administration of the DN13‐DN1 nanobody in 5xFAD mice reduces mGluR2 expression in the cortex, and this reduction is not linked to an increased neuronal loss.

## 3 Discussion

This study identifies a receptor‐specific mechanism by which mGluR2, but not mGluR3, promotes amyloidogenic processing of APP and contributes to AD pathology. Using a combination of biochemical, cellular, and in vivo approaches, we show that activation of mGluR2 increases Aβ_1–42_ and sAPPβ production via G_i/o_ signaling pathways and enhances APP internalization, thereby promoting its cleavage by β‐secretase. Accordingly, chronic administration of the mGluR2 nanobody DN13‐DN1 in 5xFAD mice worsens cognitive deficits and increases amyloid plaque deposition. In contrast, mGluR3 forms a physical complex with APP that appears to protect it from amyloidogenic processing. Co‐internalization of the mGluR3‐APP complex reroutes APP trafficking towards recycling endosomes, protecting it from β‐cleavage in late endosomes. Unlike mGluR2, our data show that activation of mGluR3 promotes sAPPα production, dependent of G_i/o_ activation, favoring non‐amyloidogenic APP processing [48]. Our findings offer a mechanistic explanation for previously inconsistent results on group II mGluR involvement in AD [17, 18] and suggest that mGluR2 and mGluR3 play fundamentally divergent roles in disease progression.

At the mechanistic level, our findings support a model in which mGluR2 activation increases APP internalization through a G_i/o_‐dependent pathway. This process does not involve β‐arrestin recruitment [49] as described for other GPCRs [50], increasing APP internalization and its β‐processing [37, 51]. Once internalized, APP is trafficked to early endosomes, where it becomes accessible to BACE1 and undergoes amyloidogenic cleavage [52, 53]. This process is consistent with previous studies showing that GPCR‐mediated endocytosis can modulate APP processing and Aβ production. Indeed, GPCR‐secretase complexes have been shown to modulate β‑ and γ‑secretase activity and Aβ generation by mediating their co‑endocytic sorting to regulate APP proteolysis and generate Aβ peptides [54].

In contrast, mGluR3 and APP direct interaction appears to prevent its routing to amyloidogenic compartments. This interaction likely retains APP at the plasma membrane and when internalized directs it toward recycling endosomes [55, 56]. Consistent with this mechanism, direct interaction between GABA_B_ and APP has been shown to reduce Aβ production by preventing APP's amyloidogenic trafficking [30]. The protective effect of mGluR3 is further supported by the observation that its activation increases sAPPα secretion, favoring the non‐amyloidogenic pathway. This aligns with previous findings showing that astroglial mGluR3 activation promotes α‐secretase cleavage of APP, reducing Aβ production [48]. However, we cannot exclude that APP mutations linked to AD in the 5xFAD mouse model used, mainly found in the transmembrane domain [40], could affect the mGluR3‐APP interaction. Finally, these distinct trafficking outcomes of APP when associated with mGluR2 or mGluR3 provide a mechanistic basis for their divergent effects on Aβ production. Consistently, domain‐swapping experiments between the C‐terminal regions of mGluR2 and mGluR3 partially reversed their APP‐binding profiles, underscoring the importance of the mGluR C‐terminal region in dictating APP fate.

Our in vivo data further establishes a causal link between mGluR2 activation and AD‐like pathology. Chronic administration of the DN13‐DN1 nanobody to 5xFAD mice significantly worsened spatial and recognition memory deficits and led to a marked increase in amyloid plaque burden, particularly in the hippocampus. In parallel, we observed a reduction of mGluR2 expression in the cortex of 5xFAD mice, possibly reflecting the selective vulnerability of mGluR2‐expressing neurons to Aβ‐mediated toxicity. This reduced expression was exacerbated upon chronic activation of mGluR2, and it is not linked to increased neurodegeneration in the cortex, but may instead reflect receptor downregulation. Indeed, some studies have reported epigenetic modifications affecting mGluR2 (Grm2) expression [45, 57, 58, 59], even though the context is different from that in our study. The hippocampus, which exhibited a greater increase in amyloid plaques compared to the cortex, may be more susceptible to mGluR2‐mediated Aβ overproduction due to differences in glial activation and amyloid clearance mechanisms. Increased astrogliosis and microgliosis in the cortex of DN13‐DN1‐treated 5xFAD mice, associated to decreased expression of mGluR2, may contribute to the limited plaque accumulation in this region compared to the hippocampus. These region‐specific differences in the pathology and receptor expression align with known variations in receptor distribution [60, 61, 62] and highlight the complex interplay between neuronal circuits, glutamatergic signaling, and amyloid deposition in AD.

Interestingly, chronic activation of mGluR2 selectively increases Aβ_1‐42_ levels, without changes in Aβ_1‐40_, suggesting a change in γ‐secretase processivity rather than a modification of β‐secretase activity. γ‐secretase cleaves APP's C‐terminal fragment (C99) sequentially, and subtle alterations in its complex composition or membrane environment can selectively modulate the ratio between Aβ_1‐42_/Aβ_1‐40_ [63]. Some GPCRs, such as the δ opioid receptor or GPR3, have been implicated in the regulation of APP proteolysis and Aβ generation [37, 54, 64]. Our data support a model where mGluR2 activation promotes APP and C99 trafficking to endosomal compartments enriched in Aβ_1‐42_‐biased γ‐secretase activity. Mechanistically, mGluR2 signaling via G_i/o_ protein reduces cAMP/PKA activity, which has been linked to slower γ‐secretase processivity and increased Aβ_1‐42_ production [65, 66]. Additionally, mGluR2‐mediated PKC activation and ERK/MAPK signaling may further modulate γ‐secretase processivity [65, 66] and APP endocytosis, potentially redirecting APP to early endosomes [67, 68, 69]. These mechanisms warrant further investigation to elucidate the precise pathways underlying the observed shift in Aβ speciation.

Importantly, this work provides a rational basis for resolving conflicting results from previous studies regarding the group II mGluR in AD. For example, non‐selectively orthosteric agonists of mGluR2 and mGluR3 have been reported to exert neuroprotective effects in mixed neuron‐astrocyte cultures [17], while other studies have shown an increase of Aβ production in synaptosomes [18]. This discrepancy can be attributed to the opposing effects of mGluR2 and mGluR3 activation, which had not previously been clearly delineated due to the lack of subtype‐specific pharmacological tools [70]. The DN13‐DN1 nanobody overcomes this limitation offering precise modulation of mGluR2 in vivo, and our results suggest that selective inhibition of mGluR2, while sparing mGluR3, may be a more effective therapeutic strategy for reducing Aβ burden and preserving cognitive function.

**FIGURE 6:**
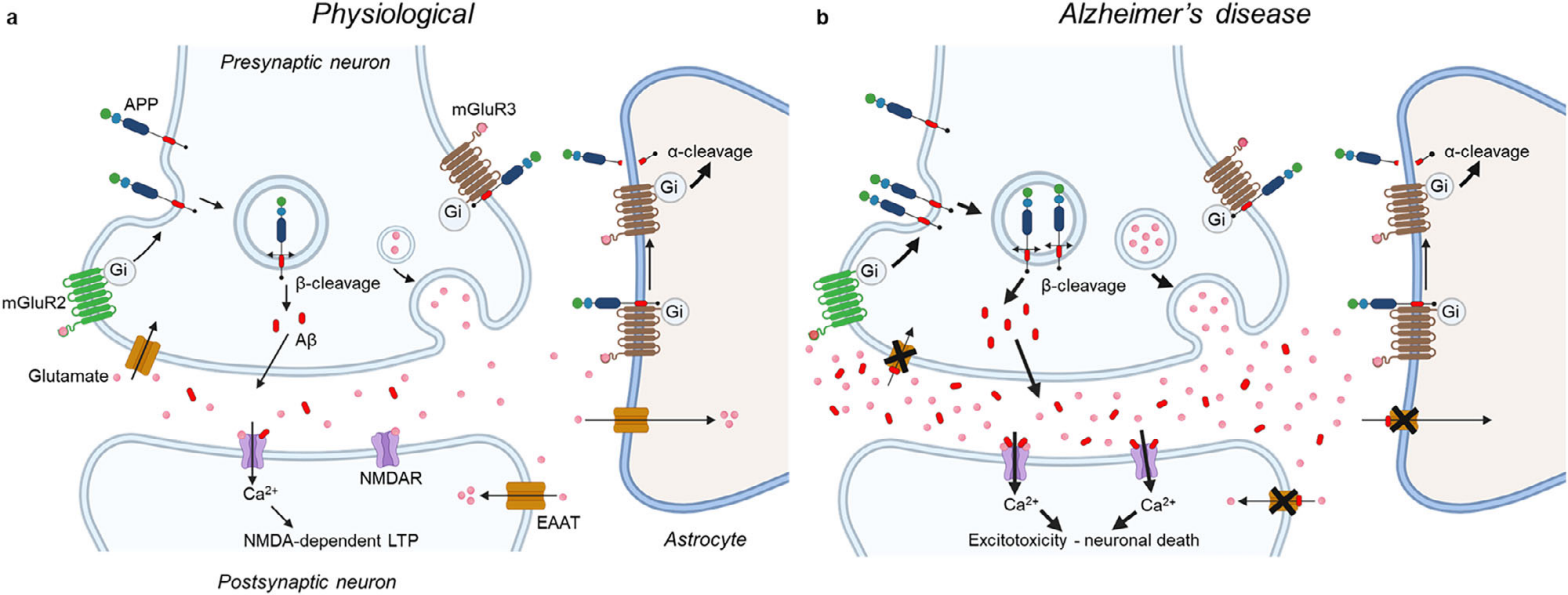
Proposed mechanisms for mGluR2 and mGluR3 in APP processing under physiological and pathological conditions. (a) Under physiological conditions, glutamate released from presynaptic terminals activates mGluR2, promoting internalization of APP. In endosomes, APP undergoes β‐secretase cleavage, generating Aβ peptides. These physiological Aβ levels potentiate NMDA receptor function and support NMDA‐dependent long‐term potentiation (LTP). mGluR3 interacts with APP, reducing its availability for β‐secretase processing and thereby limiting Aβ peptides. In astrocytes, mGluR3 activation promotes the non‐amyloidogenic APP cleavage by α‐secretases, further decreasing Aβ generation. We hypothesize that neuronal mGluR3 similarly facilitates α‐processing of APP. (b) Under pathological conditions such as AD, excessive glutamate release leads to prolonged activation of mGluR2, enhancing APP internalization and β‐cleavage, thereby increasing synaptic Aβ production. Elevated Aβ levels reinforce NMDA receptor potentiation, contributing to excitotoxicity and neuronal death. Aβ also disrupts glutamate clearance by inhibiting EAAT transporters, exacerbating synaptic glutamate accumulation. While the role of mGluR3 remains less well‐characterized in AD, we hypothesize that it continues to promote α‐cleavage of APP, offering potential neuroprotective effects. Created with BioRender.com.

These results also support the concept of the dual nature of Aβ in the central nervous system. At low concentrations, Aβ_1‐42_ may act as a neuromodulator that supports synaptic plasticity via long‐term potentiation (LTP) and memory formation [71, 72, 73], reflecting its beneficial effects under normal conditions (Figure 6a). However, under pathological conditions, such as those modeled in 5xFAD mice, excessive glutamatergic activity and chronic mGluR2 activation drive overproduction of Aβ, initiating a feedback loop that amplifies synaptic dysfunction (Figure 6b). Elevated extracellular Aβ inhibits astrocytic glutamate uptake, further increasing glutamate levels and perpetuating excitotoxicity and Aβ release [74, 75]. Our findings position mGluR2 as a molecular amplifier of this vicious cycle, linking synaptic activity to pathological APP processing.

Our results could complement ongoing therapeutic strategies focused on Aβ clearance. Monoclonal antibodies such as aducanumab, lecanemab, and donanemab aim to remove plaques and reduce soluble Aβ species [8, 9, 10, 11], but their efficacy remains modest, and their use is limited by adverse events such as ARIA [13, 76]. Moreover, with these treatments, plaque clearance occurs relatively late in the disease course, when significant synaptic and neuronal loss have already occurred [77]. In contrast, targeting Aβ production upstream, by modulating APP processing or receptor‐mediated trafficking, may prevent the accumulation of toxic Aβ species before irreversible damage ensues [78, 79, 80]. Our findings suggest that mGluR2 inhibition, potentially via nanobody‐based negative allosteric modulators, could complement or even precede Aβ‐targeting immunotherapies to achieve greater neuroprotection.

Finally, our study highlights the therapeutic potential of nanobody‐based approaches for brain diseases [23]. DN13‐DN1 is one of the first nanobodies shown to modulate a specific GPCR in the brain with high target selectivity and functional consequences on disease progression [22]. Nanobodies, by virtue of their small size, low immunogenicity [20], and high tissue penetration [21, 81, 82], overcome several limitations of conventional monoclonal antibodies, particularly for central nervous system applications. Their ability to enter the brain, either passively [22] or via receptor‐mediated mechanisms [21, 83], and engage targets such as neuroreceptors, traditionally considered inaccessible to biologics, makes nanobodies promising candidates for next‐generation immunotherapies [19, 22, 23]. Furthermore, nanobodies can be engineered for agonist, antagonist, or allosteric modulator activity, enabling precise pharmacological modulation of targets such as GPCRs [22, 24, 25, 26]. While most nanobodies in the AD field have targeted aggregates of Aβ or Tau [84, 85, 86, 87, 88, 89, 90], our work demonstrates that nanobodies can also be harnessed to modulate upstream signaling pathways involved in Aβ production. Notably, our present study shows no apparent toxicity upon chronic administration.

## 4 Conclusion

This study establishes mGluR2 as a key contributor to Aβ overproduction and AD progression, distinct from the protective role of mGluR3. Using a selective nanobody as a pharmacological agent, we uncover mechanistic insights into how mGluR2 modulates APP processing and demonstrate, in a mouse model of AD, the pathological consequences of its chronic activation. These findings identify mGluR2 as a promising therapeutic target and support the development of nanobody‐based modulators to precisely and safely regulate GPCR function in the context of neurodegenerative diseases.

## 5 Experimental Section

### 5.1 Reagents

LY379268, LY341495, quisqualate, and L‐AP4 were purchased from Tocris (Bio‐techne, Lille, France), pertussis toxin was from Merck‐Millipore (Guyancourt, France), lipofectamine 2000 was from Thermo Fisher Scientific (Invitrogen, Illkirch, France), Triton X100, thioflavin T, and guanidine hydrochloride were from Sigma‐Aldrich (Saint Quentin Fallavier, France). Tag‐lite Snap‐Lumi4‐Tb labeling reagent (#SSNPTBD), Tag‐lite Halo‐Lumi4‐Tb labeling reagent (#SHALOTBC), Tag‐lite Halo‐red labeling reagent (#SHALOREDE), Tag‐lite Snap/Clip labeling medium (#LABMED), HTRF^TM^ Anti‐6His mAb d2‐Conjugate (#61HISDLA), HTRFTM IP‐One Gq detection kit (#62IPAPEB), HTRF^TM^ human amyloid‐β_1‐42_ detection kit (#62B42PEG), HTRF^TM^ human Amyloid‐β_1‐40_ detection kit (#62B40PEG), HTRF^TM^ d2‐NHS (#65D2SABB), HTRF^TM^ Lumi4‐Tb‐NHS (#65TBSABB) and HTRF^TM^ low volume 96‐well white plates (#66PL96001) were from Revvity. Human sAPPα (#CSB‐EQ027464HU) and human sAPPβ (abx352219) Elisa assays were from CliniSciences (Nanterre, France).

### 5.2 Production and Purification of Nanobodies

Plasmid constructs of the DN1 and DN13‐DN1 nanobodies were already reported [22, 24]. Briefly, plasmids encoding the DN1 and DN13‐DN1 nanobodies were transformed in E. coli BL21DE3 strain (Life Technologies, Villebon‐sur‐Yvette, France). Then, 10 mL of preculture was added in 1 L of LB and was incubated until reaching an OD_600_ of 0.6−0.7. The nanobody expression was induced with 1 mM isopropyl β‐D‐1‐thiogalactopyranoside, and bacteria were grown overnight at 28°C. Bacteria were collected, resuspended in ice‐cold TES buffer (0.2 M Tris, 0.5 mM EDTA, 0.5 M sucrose, pH 8) to induce the periplasmic lysis. The His‐tagged nanobodies were purified by using Ni‐NTA purification column (Qiagen, Hilden, Germany) followed by a desalting step using disposable PD‐10 desalting columns (Cytiva, USA), and endotoxins were removed using Proteus NoEndo S (Generon, Slough, UK).

### 5.3 Selectivity of DN13‐DN1 Nanobody for mGlu Receptors

Human embryonic kidney 293 (HEK293) T‐type cells were grown in Dulbecco's Modified Eagle's Medium (DMEM, Life Technologies) supplemented with 10% (v/v) heat‐inactivated fetal bovine serum (FBS, Sigma‐Aldrich) and maintained in a humidified atmosphere containing 5% CO_2_ at 37°C. Absence of mycoplasma was assessed every month using the MycoAlert mycoplasma detection kit (LT07‐318, Lonza, Amboise, France). Cells were co‐transfected to express human Snap‐tagged mGluRs (mGluR1 to mGluR8) and the high‐affinity glutamate transporter EAAC1 by lipofection (Lipofectamine 2000) according to the manufacturer's instructions. Cells were plated in 96‐well plates (Grenier Bio‐One) coated with polyornithine at 10^5^ cells per well and cultured overnight at 37°C with 5% CO_2_. Twenty‐four hours after transfection, cells were incubated with DMEM‐GlutaMAX for 1 h at 37°C. Cells were then labeled with 100 nM Tag‐lite Snap‐Lumi4‐Tb labeling reagent in Tag‐lite Snap/Clip labeling medium for 1 h at 37°C and then washed three times with Tag‐lite Snap/Clip labeling medium. To determine the selectivity of DN13‐DN1 for the mGluR1–mGluR8 receptors, 100 nM of nanobody and 200 nM HTRF^TM^ Anti‐6His mAb d2‐Conjugate were applied on the Tb‐labelled cells. We also added on the cells, either agonists (1 µM of quisqualic acid for mGluR1 and mGluR5; 1 µM of LY379268 for mGluR2 and mGluR3; 10 µM of L‐AP4 for mGluR4, mGluR6, mGluR7, and mGluR8), antagonist (10 µM LY341495), or buffer, all diluted in Tag‐lite Snap/Clip labeling medium. After an overnight incubation at 22°C, the TR‐FRET signal was determined by measuring the sensitized acceptor emission (665 nm) and terbium donor emission (620 nm) using a 50 µs delay and 450 µs integration time upon excitation at 337 nm on a Pherastar FS platereader (BMG LabTech, Ortenberg, Germany). The TR‐FRET ratio was calculated as emission at 665 nm/emission at 620 nm x 10^4^.

### 5.4 Inositol‐Phosphate 1 (IP_1_) Measurements

HEK293T cells were co‐transfected with Snap‐tagged human mGluR2 (^ST^mGluR2), the chimeric G protein Gqi9, and EAAC1 by electroporation, as previously described [22]. Cells were transferred at 10^5^ cells per well in a black 96‐well plate and incubated at 37°C. Twenty‐four hours after transfection, cells were incubated with DMEM‐GlutaMAX for 1 h at 37°C, washed and stimulated with 0.88 nM of LY379268 (EC_20_) and a range of concentrations of the DN13‐DN1 and DN1 nanobodies and incubated for 30 min at 37°C. The measurement of IP_1_ accumulation was determined using the HTRFTM IP‐One Gq detection kit, according to the manufacturer's recommendation. IP‐One kit is based on a competitive format involving a monoclonal anti‐IP_1_ europium cryptate (donor) and an IP_1_ coupled to d2 (acceptor). Endogenous IP_1_ produced by cells competes with d2‐labeled IP_1_ for binding to this monoclonal anti‐IP_1_ europium cryptate, resulting in a reduced TR‐FRET signal.

### 5.5 Cell Surface Expression of Transfected mGluRs in HEK293 Cells

For all the experiments using HEK293T cells transfected, cell surface expression was performed in a 96‐well plate (Greiner bio‐one, PS, F‐bottom), with 10^5^ cells per well. Twenty‐four hours after transfection, cells were incubated with DMEM‐GlutaMAX for 1 h at 37°C and then with 100 nM of Tag‐lite Snap‐Lumi4‐Tb labeling reagent in Tag‐lite Snap/Clip labeling medium for 1 h at 37°C, to reveal expression of mGluRs. The cell surface relative expression of the different receptors was measured using the terbium donor emission at 620 nm, upon excitation at 337 nm on a Pherastar FS plate ‐reader.

### 5.6 Production and Secretion of Aβ_1‐42_ Peptides Assayed by TR‐FRET and of sAPPα and sAPPβ Assayed by ELISA in HEK293 Cells

HEK293T cells were used to measure the production and secretion of Aβ_1‐42_ peptides, *sAPPα, and sAPPβ* upon activation of mGluR2 or mGluR3 receptors. Cells were transfected by electroporation with plasmids coding for either Snap‐tagged human mGlu2 receptors (^ST^mGluR2) or human mGluR3 (^ST^mGluR3) alone, or co‐transfected with the Halo‐tagged human APP_695_ construct (^HT^APP). Cells were also co‐transfected with the high‐affinity glutamate transporter EAAC1. In each condition, 20 × 10^6^ electroporated cells were transferred into a 6‐well plate (Corning, Fisher Scientific) coated with polyornithine, 3 × 10^6^ cells per well. Mock cells were transfected with empty plasmids. Twenty‐four hours after transfection, cells were incubated with DMEM‐GlutaMAX at 37°C for 1 h, then medium was removed and cells were incubated with either PBS; agonist LY379268 (5 µM) alone, or with a pretreatment with antagonist LY341495 (10 µM), or with pertussis toxin (PTX, 0.2 µg/mL); DN13‐DN1 nanobody (200 nM); or with the EC_20_ of LY379268 (0.5 nM) alone, or combined with DN13‐DN1. After 12 h of treatment, the cell supernatant was collected and centrifuged for 10 min at 1000 g to remove cell debris. Supernatants were supplemented with a protease inhibitor cocktail (Complete ultra, Roche, Sigma‐Aldrich) and stored at −80°C until use. The human Aβ_1‐42_ peptides were assayed using the HTRF^TM^ human amyloid‐β_1‐42_ detection kit. HTRF^TM^ signal was determined by measuring the sensitized acceptor emission (665 nm) and Europium donor emission (620 nm) using a 50 µs delay and 450 µs integration time upon excitation at 337 nm on a Pherastar FS plate reader (BMG LabTech). Then, the acceptor/donor ratio (665 nm/620 nm × 10^4^) was calculated and converted into pg/mL values according to the standard curve of the assay. Human sAPPα and sAPPβ fragment levels in the supernatant were assayed using ELISA following the manufacturer's protocol. The absorbance at 450 nm was measured using an Infinite M200 (Tecan). All values obtained were normalized by the Mock condition and expressed as a fold of basal. In parallel, the relative expression of ^ST^mGluRs and ^HT^APP was measured using Tag‐lite Snap or Halo‐Lumi4‐Tb labeling reagents, respectively, as described above.

### 5.7 TR‐FRET Saturation Assay

TR‐FRET saturation assays were performed as previously described [29] to determine the potential association of APP with mGluR2 or mGluR3. Briefly, HEK293T cells were co‐transfected by lipofection (Lipofectamine 2000) with a constant amount of plasmid encoding ^ST^APP, ^ST^mGluR2, ^ST^GB1a‐ASA or ^ST^mGluR3 (20 ng), and increasing amounts of ^HT^mGluR2, ^HT^mGluR3, ^HT^mGluR5, ^HT^APP, ^HT^mGluR3, ^HT^mGluR3 ΔCtem (Del829‐879), ^HT^mGluR3 ECD‐GPI (Del570‐879), ^HT^APP ΔCtem (Del648‐695), ^HT^mGluR2‐C3 Cterm or ^HT^mGluR3‐C2 Cterm (from 0 to 160 ng). Cells were plated in 96‐well plates coated with polyornithine at 10^5^ cells per well. Twenty‐four hours after transfection, cells were incubated with DMEM‐GlutaMAX 1 h at 37°C and then with either 100 nM of Tag‐lite Snap‐Lumi4‐Tb labeling reagent to measure ^ST^receptors’ expression (referred to as Exp ST), Tag‐lite Halo‐Lumi4‐Tb labeling reagent to measure ^HT^receptors (Exp HT), or with a combination of 100 nM of Tag‐lite Snap‐Lumi4‐Tb labeling reagent and 100 nM of Tag‐lite Halo‐red labeling reagent to measure the FRET signal. Cells were then washed three times with Tag‐lite Snap/Clip labeling medium, and fluorescence as well as TR‐FRET were read using an Infinite F500 spectrofluorometer (Tecan).

### 5.8 Protein Extraction and Co‐Immunoprecipitation Assays

Cortex tissues from WT mice were rapidly dissected and homogenized in an ice‐cold lysis buffer (150 mM sodium chloride, 50 mM tris‐HCl pH 7.5, 2 mM EDTA, 2 mM EGTA, 1 mM sodium orthovanadate, 50 mM sodium fluoride, 25 mM sodium ß‐glycerophosphate, 5 mM sodium pyrophosphate, 0.1% SDS, 2% Triton X‐100, 2% LMNG and complete protease inhibitor EDTA free (1 pellet for 20 mL; Roche Diagnostics). Lysates were centrifuged (10 min, 16.000 g) to eliminate insoluble material. Soluble proteins were quantified by bicinchoninic acid assay (BCA) method, protein assay (Sigma‐Aldrich), and equal protein amounts (1 mg) were incubated with either an anti‐mGlu2 nanobody (DN13‐DN1; 5 µg) or an anti‐mGluR3 nanobody and nickel‐nitrilotriacetic acid agarose beads. Control immunoprecipitations were performed with only the agarose beads. Experiments were performed in triplicate, each from the cortex of one mouse. Samples were centrifuged (5 min, 7000 g) and washed three times with lysis buffer containing 500 mM NaCl and 0.5 % LMNG and three times with lysis buffer containing 150 mM NaCl and 0.5 % LMNG. After reduction (β‐mercaptoethanol, 10 mM, 30 min at 37°C), samples were frozen at −80°C until use.

### 5.9 Western Blotting

Proteins were resolved by SDS polyacrylamide (4%–15%, Criterion) gel electrophoresis and transferred (Trans‐Blot Turbo, Bio‐Rad) to nitrocellulose membrane (Bio‐Rad, #1620113). Membranes were saturated with tris‐buffered saline supplemented with 0.1% Tween 20 and 5% skimmed milk and incubated with either the mouse anti‐APP (6E10 clone, 1:1000, #MA5‐51794, Thermo Fisher Scientific); the rabbit anti‐mGluR3 (1:1000, #ab166608, Abcam), or the rabbit anti‐mGluR2 (1:5000; #ab106811, Abcam), and then with anti‐rabbit or anti‐mouse HRP‐conjugated secondary antibodies (1:5000, Jackson Immunoresearch). Immunoreactivity was detected with the SuperSignal West Pico PLUS chemiluminescence substrate (Thermo Scientific) using a ChemiDoc MP Imaging System (Bio‐Rad).

### 5.10 Diffusion‐Enhanced Resonance Energy Transfer (DERET) Internalization Assay

A DERET assay [34, 35] was performed to measure the internalization in real time of the human APP_695_ upon activation of mGluR2 or mGluR3. The internalization assay was performed and adapted as previously described [34]. HEK293T cells were transfected by lipofection either with ^ST^mGluR2, ^ST^mGluR3 or ^HT^APP alone, or co‐transfected with ^ST^mGluR2 or ^ST^mGluR3 with the ^HT^APP. In all conditions, the high‐affinity glutamate transporter EAAC1 was also transfected. Cells were transferred to black non‐transparent 96‐well plates at 10^5^ cells per well. Twenty‐four hours after transfection, receptors were incubated with DMEM‐GlutaMAX 1 h at 37°C and then labeled with either 100 nM of Tag‐lite Snap‐Lumi4‐Tb labeling reagent or 100 nM of Tag‐lite Halo‐Lumi4‐Tb labeling reagent in Tag‐lite Snap/Clip labeling medium for 1.5 h at 4°C. Excess of Snap‐ or Halo‐Lumi4‐Tb substrates was removed by washing cells with Tag‐lite Snap/Clip labeling medium. The internalization assay was performed by incubating the cells with the Tag‐lite Snap/Clip labeling medium, either with PBS or with 1 µM of LY376892, and in the presence of an excess of fluorescein (25 µM). Lumi4‐Tb was excited at 337 nm, and the emission fluorescence intensities were recorded for the donor (620 nm, 1500 µs delay, 1500 µs reading time) and acceptor (520 nm, 150 µs delay, 400 µs reading time) using a pherastar FS microplate reader. Both intensities (620 nm and 520 nm) were measured every 10 min for 90 min. The ratio of 620 nm/520 nm was then calculated for each time point and multiplied by 10^4^. In parallel, the relative expression of ^ST^mGluRs and ^HT^APP was measured, as described above. The same procedure was performed for β‐arrestin knockout cells [38] (kindly provided by A. Inoue, Tohoku University, Sendai, Japan) that were transfected by lipofection either with ^ST^mGluR2 or ^HT^APP alone, or co‐transfected with ^ST^mGluR2 and ^HT^APP. Cells were also co‐transfected with the high‐affinity glutamate transporter EAAC1.

### 5.11 Immunofluorescence, Confocal Microscopy, and Image Data Analysis of APP Trafficking in Early and Recycling Endosomes in HEK293 Cells

HEK293T cells were transiently co‐transfected with ^HT^APP, ^ST^mGluR3, and EAAC1 using Lipofectamine 2000. The transfection mixture and lipofectamine 2000 were added to cells cultured with DMEM in a 6‐well plate. After 24 h, ^HT^APP and ^ST^mGluR3 were labeled with 100 nM Halo‐tag Alexa Fluor 488 and 100 nM Snap‐tag Alexa Fluor 594 in DMEM‐GlutaMAX, supplied with LY341495 (10 µM) for 1 h at 37°C, 5% CO_2_. Cells were then washed 3 times with PBS. Cells were then treated with the mGluR3 agonist LY379268 (5 µM) or with PBS in DMEM‐GlutaMAX, 30 min before fixation. Cells were then fixed with 4% paraformaldehyde in PBS and permeabilized with saponin (Sigma‐Aldrich) in PBS. Cells were incubated with either a rabbit anti‐Rab5 (1:300; #3547, Cell Signaling Technology) or a rabbit anti‐Rab11 (1:300; ab128913, Abcam), then with a goat anti‐rabbit Alexa Fluor 647 antibody (1:1000; #A‐21245, ThermoFisher Scientific) and mounted with Fluoroshield containing DAPI. Immunofluorescence images were acquired with a Leica SP8 UV/visible laser confocal microscope (objective 63×; numerical aperture of 1.4), using Leica LAS X software. For image analysis, deconvolution software was applied to remove out‐of‐focus information using Huygens Essential. Images and composite figures were prepared using Image J, and colocalization analysis was performed using the BIOP‐JaCoP plugin (revamped version by BioImaging and Optics Platform, EPFL).

### 5.12 Ethics

This project follows the specific French national guidelines on animal experimentation and well‐being according to the European Directive 2010/63/EU. This project was approved by the French National Ethic Committee for Animal Experimentation and the French Ministry of Agriculture under the number APAFIS #28773‐2020121811289735v4. Animals were housed under a 12 h light/12 h dark cycle and at 23 ± 2°C. Animals had free access to water and food and were fed under a standard chow diet (A03) (SAFE Diets, Augy, France).

### 5.13 Animals

5xFAD mice overexpress the human APP with the Swedish (K670N, M671L), Florida (I716V) and London (V717I) mutations and the human presenilin1 (PS1) gene harboring the M146L and L286V mutations [40]. These transgenes are regulated by the neuronal‐specific elements of the mouse Thy1 promoter. 5xFAD heterozygous transgenic mice were used for the experiments, and WT littermates as controls. All animals were genotyped by qPCR using tail genomic DNA [40]. To study the effect of a chronic positive modulation of mGluR2 in vivo, a nanobody specific to mGlu2 receptors and exhibiting PAM effects [22] (DN13‐DN1) was used. As a negative control, we used the DN1 nanobody [24], with a neutral effect on mGluR2 activation. In this study, male mice were chronically treated with 10 mg/kg of either DN13‐DN1 (n = 20 WT and n = 21 5xFAD) or DN1 (*n* = 18 WT and *n* = 16 5xFAD), by intraperitoneal route, once a week, for 22 weeks. Control mice were treated with an equivalent volume of PBS (*n* = 16 WT and *n* = 22 5xFAD). Mice were followed up and weighed every week. After treatment, mice underwent several behavioral tests and were sacrificed.

### 5.14 Behavioral Tests

One week before behavioral tests, mice were handled every day by the operator. The number of mice used in each group for behavioral tests is: DN13‐DN1 nanobody treatment (*n* = 17 WT and *n* = 17 5xFAD), DN1 treatment (*n* = 15 WT and *n* = 11 5xFAD), and PBS (*n* = 16 WT and *n* = 22 5xFAD).

#### 5.14.1 Nest Building

Animals housed in group were placed in individual testing cages and received 3 g of nestlet for an overnight nest building. The next morning, nest quality was scored, and non‐shredded nestlet pieces were weighed. Scoring of the nest quality was performed using a 5‐point nest‐rating scale, as previously described [91].

#### 5.14.2 Marble‐Burying

A plastic cage (width: 23.4 cm, length: 37.3 cm, height: 14.0 cm) was filled with 5 cm of bedding, and 18 marbles were placed on top of this bedding, 3 marbles in 6 rows. Each mouse was placed in an individual testing cage and was left with the marbles for 30 min. Then, the mouse was removed, and the number of buried marbles was counted. We considered a marble buried when at least two‐thirds of the marble was covered of bedding as previously described in this test [92].

#### 5.14.3 T‐maze

The T‐maze is composed of one main runway (width: 10 cm, length: 50 cm, height: 14.0 cm) connected to two side arms (width: 10 cm, length: 20 cm, height: 14.0 cm) made of acrylic plastic painted in light grey color. During the T‐maze spatial habituation, each mouse was allowed to explore for 5 min the main runway and one of the side arms. Mice were placed back to their home cages for 25 min. Then, the second (novel) side arm was opened, and the ambulation of mice in each side arm was observed for 5 min. The number of entries in each side arms was quantified, and the discrimination index was calculated as “(novel—familiar) / (familiar + novel)”.

#### 5.14.4 Novel Object Recognition

This behavioral test was performed as previously described [93]. In this study, white acrylic boxes (width: 28 cm, length: 41 cm, height: 28 cm) and three distinct objects with different shapes were used. During the behavioral period, the room brightness was dimmed (30 lux) and bedding was spread on the floor of the apparatus to reduce anxiety. Each day, prior to the behavioral test, mice were familiarized with the testing room for at least 1 h. On the first day, all mice were exposed to the empty arena for 5 min for habituation. The next day, during the familiarization phase, all mice were exposed to two identical objects for 5 min. Four hours after the familiarization, one object was kept as a familiar object, while the other was replaced by a novel object. Mice were allowed to explore the objects for 5 min. The same procedure was performed 24 h after familiarization by using a different novel object than the one used at 4 h.

For the habituation trials, the mean velocity, the time spent in the center of the arena, the total distance traveled, and the time of immobility were measured using EthoTrack software (v. 3.11.0, Innovation Net, Tiranges, France). For the other trials, a discrimination index using the exploration times ([novel—familiar] / [familiar + novel]) was calculated. Direct contact or sniffing behavior close to objects (< 1 cm) was defined as object exploration, while climbing and digging were not considered. Mice showing a preference for one of the familiar objects or having a total exploration time of the objects of less than 6 s during trials were excluded from behavioral analyses. Two independent quantifications of the discrimination indexes were performed blindly, and the same trends were observed for the two sets of values obtained. Results from the two independent quantifications were averaged for each mouse.

### 5.15 Culling of Mice and Tissue Collection

This procedure was performed as previously described [94]. Briefly, mice were anesthetized (16% ketamine (Imalgene 500, Merial), 4% xylazine (Rompun 2%, Bayer) in NaCl solution at 0.9%) and sacrificed by an intracardial perfusion of PBS. Brains were removed and cut along the sagittal axis. Left hemispheres were directly frozen in liquid nitrogen and stored at ‐80°C (for biochemical analyses), and right hemispheres were in part fixed in a 4% paraformaldehyde solution (PFA, Sigma‐Aldrich) overnight at 4°C (for immunolabeling). Fixed hemibrains were rinsed with PBS and incubated in a 30% sucrose solution for 4 days at 4°C. Brains were then included in O.C.T. (Tissue‐Tek, Sakura Finetek, Villeneuve‐d'Ascq, France), quickly frozen on acetone chilled on dry ice, and conserved at −80°C.

The other part of the right hemisphere was collected to perform TR‐FRET experiments as described [22, 26]. For each hemibrain, different regions were punched: cortex, hippocampus, cerebellum, and the midbrain. Samples were collected in a 1.5 mL cryogenic tube (Thermo Fisher Scientific) in 1 mL cold cryopreservation medium (DMEM‐GlutaMAX supplemented with 10% FBS and 10% DMSO), frozen at −80°C in a freezing box, and kept at −80°C until use.

In parallel, in 2 WT mice from each group (PBS, DN13‐DN1 and DN1), 27 organs were collected (eyes with optical nerve, harderian glands, salivary glands, stomach, pancreas, intestines (duodenum, ileum, jejunum, and colon), cecum, testes, epididymis, seminal vesicles, prostate, urinary bladder, kidneys, adrenal glands, white adipose tissue, spleen, liver, heart, aorta, lungs, esophagus, trachea, bone with bone marrow, lymph nodes, sciatic nerve, skeletal muscle, and skin). All the samples were incubated overnight in a 4% PFA solution at 4°C, washed with PBS, and kept in 70% ethanol. All samples were processed for anatomopathological studies according to the international RITA procedure for toxicological studies [95, 96, 97]. Samples were dehydrated in graded ethanol and embedded in paraffin.

### 5.16 Brain Extracts Preparation

Frozen left hemispheres of 5xFAD (22 vehicle, 17 DN13‐DN1 and 11 DN1) were thawed, weighed, and homogenized with a Potter in 20% wt/vol in a tris‐saline buffer (20 mM Tris‐HCl, pH 7.4; 150 mM NaCl) containing a protease inhibitor cocktail (Complete ultra). Homogenates were then ultracentrifuged at 355 000 g at 4°C for 20 min (TLA 120.1 rotor, Beckman Optima TLX ultracentrifuge) and supernatants (referred as “soluble fraction”) were collected and kept at −80°C, until use. Pellets were resuspended by brief sonication in 6 M guanidine HCl and 20 mM Tris‐HCl (pH 7.6) buffer and ultracentrifuged at 227 000 g at 4°C for 20 min. Supernatants obtained (referred as “insoluble fraction”) were collected and stored at −80°C. For WT mice hemibrains (12 vehicle, 12 DN13‐DN1, and 10 DN1), the same procedure was performed.

### 5.17 Amyloid‐β Assays

The HTRF^TM^ human Amyloid‐β_1‐42_ detection kit and the HTRF^TM^ human Amyloid‐β_1‐40_ detection kit were used according to the manufacturer's instructions. FRET signal was determined as explained above, on the soluble and insoluble fractions obtained from brain extracts. HTRF signal was determined by measuring the sensitized acceptor emission (665 nm) and Europium donor emission (620 nm) using a 50 µs delay and 450 µs integration time upon excitation at 337 nm on a Pherastar FS plate‐reader (BMG LabTech). Then, the acceptor/donor ratio (665 nm/620 nm × 10^4^) was calculated and converted into pg/mL values according to the standard curve of the assay. Mouse Aβ_1‐42_ was assayed using an Elisa kit from Thermo Fisher Scientific (#KMB3441). Reactions were read at 450 nm using an Infinite M200 (Tecan). All values were normalized according to their protein concentration determined by the BCA method protein assay (Sigma‐Aldrich). BCA OD at 562 nm was measured using an Infinite M200 (Tecan).

### 5.18 Aggregate Staining and Quantification

Frozen hemibrains included in OCT were mounted on a cryostat (Leica) and 20 µm coronal sections were performed and conserved at ‐20°C in an antifreeze solution. Brain tissue sections were incubated with a thioflavin T at 0.01% for 10 min [94]. Sections were washed with an 80% ethanol solution, rinsed with distilled water, and mounted using Fluoroshield containing DAPI to stain nuclei (Sigma‐Aldrich). All sections were visualized using a slide scanner Axio scan Z1 microscope (Zeiss, Jena, Germany) by performing a full‐section mosaic with a 20x enlargement. Images were analyzed with Fiji software (version 2.0; National Institutes of Health) and the number and the surface of amyloid aggregates were quantified as previously described [94]. The number of brains used per group to quantify the amyloid plaques is the following: DN13‐DN1 (*n* = 6 WT and *n* = 7 5xFAD), DN1 (*n* = 7 WT and *n* = 6 5xFAD), and vehicle (*n* = 8 WT and *n* = 8 5xFAD).

### 5.19 Immunofluorescence and Quantifications

Neuroinflammation was assessed with a mouse anti‐GFAP antibody (1:1.000; Sigma‐Aldrich) for astrogliosis or with a rabbit polyclonal anti‐IBA1 antibody (1:750; Wako Chemicals) for microgliosis. Briefly, brain tissue sections were blocked 1 h at room temperature and incubated with one of the primary antibodies overnight at 4°C. Then, sections were washed and incubated with secondary antibodies, a goat anti‐mouse Cy3 (1:1.000) or a goat anti‐rabbit Alexa Fluor 488 (1:1.000) for 2 h at room temperature, and mounted with Fluoroshield containing DAPI. All sections were acquired using a slide scanner Axio scan Z1 microscope by performing a full‐section mosaic with a 20× enlargement and quantifications were performed using Fiji software. Astrogliosis and microgliosis were evaluated by quantifying the intensity of the labeling and the area labeled in both full cortex and full hippocampus. The intensity and the area of the labeling were measured on 8‐bit images using the threshold tool of the Fiji software, and values were normalized by the surface area of the cortex or hippocampi analyzed. The same threshold parameters were used for all images.

### 5.20 Cresyl Violet Staining

Brain tissue sections were stained with a 0.5 % cresyl violet solution for 5 min, washed, and then dehydrated sequentially in 70 %, 95 %, and 100 % ethanol solutions for 2 min each. Brain sections were cleared in xylene for 2 min and mounted with Pertex after air drying. All sections were visualized using a slide scanner Axio scan Z1 microscope by performing a full‐section mosaic with a 20× enlargement. Images were analyzed with Fiji software and the number of viable and damaged neurons were quantified [98]. Viable neuron was characterized by properties such as exhibiting a visible nucleus and nucleolus with light purple stain in the cytoplasm, while the damaged ones by a dark purple staining of cresyl violet with cell shrinkage, inability to reveal nucleolus, and appearance of a vacuole around neuron.

### 5.21 HES Staining for Toxicological Study

The 27 samples collected from WT mice and embedded in paraffin were cut using a microtome, and 5 µm tissue sections were performed. All sections were mounted on Superfrost Plus slides, dewaxed, and stained with hematoxylin (nucleus staining), eosin (cytoplasm staining) and natural saffron (collagen fibers staining) (HES). Sections were washed, dehydrated and then coverslipped with Permount mounting medium (Fisher Scientific). All sections were visualized using a slide scanner Axio scan Z1 microscope by performing full‐section mosaic with a 20× enlargement.

### 5.22 Nanobody Coupling to Fluorophores

The mGluR2 specific nanobody DN1 was labelled as previously described [22, 26]. Briefly, the nanobody was dialyzed overnight at 4°C. Then, 250 µg of nanobody were incubated at 20°C with HTRF^TM^ d2‐NHS in 0.1 M carbonate buffer (pH 9) at a molar ratio of 6, for 45 min. In parallel, 250 µg of the dialyzed nanobody were incubated with HTRF^TM^ Lumi4‐Tb‐NHS in 50 mM phosphate buffer (pH 8) at a molar ratio of 12 for 30 min. Coupled nanobodies were then purified using a gel filtration column in 100 mM phosphate buffer at pH 7, and their concentration was determined by OD at 280 nm. After purification, the labelled nanobodies were supplemented with 0.1 % BSA and stored at −20°C, until use.

### 5.23 Relative Quantification of the mGluR2 Homodimers by TR‐FRET in Brains from WT and 5xFAD Mice

For this experiment, the number of hemibrains analyzed was as follows: DN13‐DN1 (*n* = 6 WT and *n* = 8 5xFAD), DN1 (*n* = 3 WT and *n* = 3 5xFAD), and PBS (*n* = 5 WT and *n* = 10 5xFAD). The TR‐FRET protocol was performed as described previously [22, 26]. Samples were rapidly thawed in a water bath at 37°C, and tissues were washed 2 times with DMEM‐GlutaMAX and 2 times with cold PBS. Tissues were then digested with 200 µL Versene solution (ThermoFisher) for 20 min at 20°C, and the cells were dissociated by several pipettings. Several rounds of cell dissociation in DMEM‐GlutaMAX were performed to get the maximum amount of cells. Then, dissociated cells were centrifuged at 3,000 g for 5 min, and the cell pellet was washed with 1 mL of cold PBS and resuspended in 200 µL of cold PBS. Then, 10 µL of cells were added in HTRF^TM^ low volume 96‐well white plates and incubated with 10 µL of DN1‐d2 (25 nM), 10 µL of DN1‐Tb (25 nM) and 10 µL of LY341495 (10 µM), to reach a total volume of 40 µL. Plates were incubated overnight at 22°C and the TR‐FRET signal was determined by measuring the sensitized acceptor emission (665 nm) and donor emission (620 nm) using a 50 µs delay and 450 µs integration time upon excitation at 337 nm on a Pherastar FS plate reader. In parallel, the total amount of proteins was determined by the BCA protein assay (Sigma‐Aldrich). Absorbances were measured using an Infinite M200.

### 5.24 Statistical Analysis

All data were initially analyzed on GraphPad Prism software (version 10.0; GraphPad) using Shapiro‐Wilk's normality test. When data were normally distributed (*p* > 0.05), statistical analyses were performed using parametric tests: unpaired T‐test, one‐way ANOVA (Holm‐Sidak's *post‐hoc* analysis) or the two‐way ANOVA (Holm‐Sidak's *post‐hoc* analysis). However, when data were not normally distributed (*p* < 0.05) statistical analyses were performed using a nonparametric test: Kruskal‐Wallis (Dunn's *post‐hoc* analysis). A *p*‐value of 0.05 has been defined as a demonstrating a significant difference for all statistical analyses.

## Author Contributions

P.‐A.L., L.P., J.‐P.P., V.P., J.L., and P.R. designed research. S.C. contributed to the design of mouse experiments. P.‐A.L., M.E.T., U.G.A., Y.‐L.L., M.O., M.C., G.D., J.M., S.R., J.B., S.D., F.V., P.C., J.K., and A.M. performed research experiments. P.‐A.L., C.E.P., Y.‐L.L., M.C., G.D., and J.B. analyzed the data. P.‐A.L., V.P., and P.R. wrote the paper and all authors reviewed and edited the manuscript.

## Conflicts of Interest

The authors have related patents granted under patent numbers WO2016001417A1 (J.‐P.P., P.C. and P.R.), WO2024003389A1 (J.‐P.P., P.R., P.C., J.K., and M.O.), and WO2024003390A1 (J.‐P.P., P.R., L.P., J.K., M.O., and M.E.T.). P.R., L.P. and J.‐P.P. are involved in a collaborative team between the CNRS (IGF, Montpellier) and Revvity. The remaining authors declare no competing interests.

## Supporting information




**Supporting File**: advs75218‐sup‐0001‐SuppMat.docx.

## Data Availability

All data needed to evaluate the conclusions in the paper are present in the paper and/or the Supporting Information. The mGlu2‐specific nanobody can be provided, pending scientific review of the project and a completed material transfer agreement. Request for this nanobody should be submitted to P.R. (philippe.rondard@igf.cnrs.fr).

## References

[1] G. Livingston , J. Huntley , K. Y. Liu , et al., “Dementia Prevention, Intervention, and Care: 2024 Report of the Lancet standing Commission,” Lancet 404 (2024): 572–628, 10.1016/S0140-6736(24)01296-0. 39096926

[2] C. L. Masters , R. Bateman , K. Blennow , C. C. Rowe , R. A. Sperling , and J. L. Cummings , “Alzheimer's Disease,” Nature Reviews Disease Primers 1 (2015): 15056, 10.1038/nrdp.2015.56. 27188934

[3] H. Hampel , J. Hardy , K. Blennow , et al., “The Amyloid‐β Pathway in Alzheimer's Disease,” Molecular Psychiatry 26 (2021): 5481–5503, 10.1038/s41380-021-01249-0. 34456336 PMC8758495

[4] C. R. Jack , D. A. Bennett , K. Blennow , et al., “NIA‐AA Research Framework: toward a Biological Definition of Alzheimer's Disease,” Alzheimer's & Dementia 14 (2018): 535–562, 10.1016/j.jalz.2018.02.018. PMC5958625 29653606

[5] J. A. Hardy and G. A. Higgins , “Alzheimer's Disease: the Amyloid Cascade Hypothesis,” Science 256 (1992): 184–185, 10.1126/science.1566067. 1566067

[6] K. Blennow , M. J. de Leon , and H. Zetterberg , “Alzheimer's Disease,” Lancet 368 (2006): 387–403, 10.1016/S0140-6736(06)69113-7. 16876668

[7] E. Karran and B. De Strooper , “The Amyloid Hypothesis in Alzheimer Disease: New Insights from New Therapeutics,” Nature Reviews Drug Discovery 21 (2022): 306–318, 10.1038/s41573-022-00391-w. 35177833

[8] J. Sevigny , P. Chiao , T. Bussière , et al., “The Antibody Aducanumab Reduces Aβ Plaques in Alzheimer's Disease,” Nature 537 (2016): 50–56, 10.1038/nature19323. 27582220

[9] R. B. DeMattos , J. Lu , Y. Tang , et al., “A Plaque‐Specific Antibody Clears Existing β‐amyloid Plaques in Alzheimer's Disease Mice,” Neuron 76 (2012): 908–920, 10.1016/j.neuron.2012.10.029. 23217740

[10] J. R. Sims , J. A. Zimmer , C. D. Evans , et al., “Donanemab in Early Symptomatic Alzheimer Disease,” JAMA 330 (2023): 512–527, 10.1001/jama.2023.13239. 37459141 PMC10352931

[11] M. A. Mintun , A. C. Lo , C. Duggan Evans , et al., “Donanemab in Early Alzheimer's Disease,” New England Journal of Medicine 384 (2021): 1691–1704, 10.1056/NEJMoa2100708. 33720637

[12] A. Mullard , “Controversial Alzheimer's Drug Approval Could Affect Other Diseases,” Nature 595 (2021): 162–163, 10.1038/d41586-021-01763-9. 34193994

[13] S. Salloway , S. Chalkias , F. Barkhof , et al., “Amyloid‐related Imaging Abnormalities in 2 Phase 3 Studies Evaluating aducanumab in Patients with Early Alzheimer Disease,” JAMA Neurology 79 (2022): 13–21, 10.1001/jamaneurol.2021.4161. 34807243 PMC8609465

[14] J.‐P. Pin and B. Bettler , “Organization and Functions of mGlu and GABAB Receptor Complexes,” Nature 540 (2016): 60–68, 10.1038/nature20566. 27905440

[15] F. Caraci , F. Nicoletti , and A. Copani , “Metabotropic Glutamate Receptors: the Potential for Therapeutic Applications in Alzheimer's Disease,” Current Opinion in Pharmacology 38 (2018): 1–7, 10.1016/j.coph.2017.12.001. 29278824

[16] J. P. Pin and R. Duvoisin , “The Metabotropic Glutamate Receptors: Structure and Functions,” Neuropharmacology 34 (1995): 1–26, 10.1016/0028-3908(94)00129-G. 7623957

[17] F. Caraci , G. Molinaro , G. Battaglia , et al., “Targeting Group II Metabotropic Glutamate (mGlu) Receptors for the Treatment of Psychosis Associated with Alzheimer's Disease: Selective Activation of mGlu2 Receptors Amplifies Beta‐amyloid Toxicity in Cultured Neurons, Whereas Dual Activation of mGlu2 and mGlu3 Receptors Is Neuroprotective,” Molecular Pharmacology 79 (2011): 618–626, 10.1124/mol.110.067488. 21159998

[18] S. H. Kim , P. E. Fraser , D. Westaway , P. H. S. George‐Hyslop , M. E. Ehrlich , and S. Gandy , “Group II Metabotropic Glutamate Receptor Stimulation Triggers Production and Release of Alzheimer's Amyloid β 42 from Isolated Intact Nerve Terminals,” Journal of Neuroscience 30 (2010): 3870–3875, 10.1523/JNEUROSCI.4717-09.2010. 20237257 PMC2857209

[19] I. Jovčevska and S. Muyldermans , “The Therapeutic Potential of Nanobodies,” Biodrugs 34 (2020): 11–26, 10.1007/s40259-019-00392-z. 31686399 PMC6985073

[20] C. Ackaert , N. Smiejkowska , C. Xavier , et al., “Immunogenicity Risk Profile of Nanobodies,” Frontiers in Immunology 12 (2021): 632687, 10.3389/fimmu.2021.632687. 33767701 PMC7985456

[21] M. E. Tsitokana , P.‐A. Lafon , L. Prézeau , J.‐P. Pin , and P. Rondard , “Targeting the Brain with Single‐domain Antibodies: Greater Potential Than Stated so Far?,” International Journal of Molecular Sciences 24 (2023): 2632, 10.3390/ijms24032632. 36768953 PMC9916958

[22] M. Oosterlaken , A. Rogliardo , T. Lipina , et al., “Nanobody Therapy Rescues Behavioural Deficits of NMDA Receptor Hypofunction,” Nature 645 (2025): 262–270, 10.1038/s41586-025-09265-8. 40702184 PMC12408334

[23] P.‐A. Lafon , L. Prézeau , J.‐P. Pin , and P. Rondard , “Nanobodies: a New Paradigm for Brain Disorder Therapies,” Trends in Pharmacological Sciences 46 (2025): 1049–1051, 10.1016/j.tips.2025.10.004. 41198506

[24] P. Scholler , D. Nevoltris , D. de Bundel , et al., “Allosteric Nanobodies Uncover a Role of Hippocampal mGlu2 Receptor Homodimers in Contextual Fear Consolidation,” Nature Communications 8 (2017): 1967, 10.1038/s41467-017-01489-1. PMC5719040 29213077

[25] J. Haubrich , J. Font , R. B. Quast , et al., “A Nanobody Activating Metabotropic Glutamate Receptor 4 Discriminates between Homo‐ and Heterodimers,” Proceedings of the National Academy of Sciences 118 (2021): 2105848118, 10.1073/pnas.2105848118. PMC8379968 34385321

[26] J. Meng , C. Xu , P.‐A. Lafon , et al., “Nanobody‐based Sensors Reveal a High Proportion of mGlu Heterodimers in the Brain,” Nature Chemical Biology 18 (2022): 894–903, 10.1038/s41589-022-01050-2. 35681029

[27] S. Dogra and P. J. Conn , “Metabotropic Glutamate Receptors as Emerging Targets for the Treatment of Schizophrenia,” Molecular Pharmacology 101 (2022): 275–285, 10.1124/molpharm.121.000460. 35246479 PMC9092465

[28] E. Doumazane , P. Scholler , L. Fabre , et al., “Illuminating the Activation Mechanisms and Allosteric Properties of Metabotropic Glutamate Receptors,” Proceedings of the National Academy of Sciences 110 (2013): 1416–1425, 10.1073/pnas.1215615110. PMC3625292 23487753

[29] E. Doumazane , P. Scholler , J. M. Zwier , E. Trinquet , P. Rondard , and J.‐P. Pin , “A New Approach to Analyze Cell Surface Protein Complexes Reveals Specific Heterodimeric Metabotropic Glutamate Receptors,” FASEB Journal 25 (2011): 66–77, 10.1096/fj.10-163147. 20826542

[30] M. C. Dinamarca , A. Raveh , A. Schneider , et al., “Complex Formation of APP with GABAB Receptors Links Axonal Trafficking to Amyloidogenic Processing,” Nature Communications 10 (2019): 1331, 10.1038/s41467-019-09164-3. PMC6430795 30902970

[31] C. E. Philibert , C. Disdier , P.‐A. Lafon , et al., “TrkB Receptor Interacts with mGlu2 Receptor and Mediates Antipsychotic‐Like Effects of mGlu2 Receptor Activation in the Mouse,” Science Advances 10 (2024): adg1679, 10.1126/sciadv.adg1679. PMC10816717 38277461

[32] R. G. Perez , S. Soriano , J. D. Hayes , et al., “Mutagenesis Identifies New Signals for β‐Amyloid Precursor Protein Endocytosis, Turnover, and the Generation of Secreted Fragments, Including Aβ42,” Journal of Biological Chemistry 274 (1999): 18851–18856, 10.1074/jbc.274.27.18851. 10383380

[33] J. Aow , T.‐R. Huang , Y. T. Goh , A. X. Sun , G. Thinakaran , and E. H. Koo , “Evidence for a Clathrin‐independent Endocytic Pathway for APP Internalization in the Neuronal Somatodendritic Compartment,” Cell Reports 42 (2023): 112774, 10.1016/j.celrep.2023.112774. 37450368 PMC10449584

[34] M. Cimadevila , J. Liu , D. Maurel , et al., “Non‐canonical Internalization Mechanisms of mGlu Receptors,” Cell Reports 44 (2025): 116068, 10.1016/j.celrep.2025.116068. 40748754

[35] A. Levoye , J. M. Zwier , A. Jaracz‐Ros , et al., “A Broad G Protein‐coupled Receptor Internalization Assay That Combines SNAP‐tag Labeling, Diffusion‐enhanced Resonance Energy Transfer, and a Highly Emissive Terbium Cryptate,” Frontiers in Endocrinology 6 (2015): 167, 10.3389/fendo.2015.00167. 26617570 PMC4638144

[36] C. D. Nelson and M. Sheng , “Gpr3 Stimulates Aβ Production via Interactions with APP and β‐arrestin2,” PLoS ONE 8 (2013): 74680, 10.1371/journal.pone.0074680. PMC3771882 24069330

[37] Y. Huang , T. Rafael Guimarães , N. Todd , et al., “G Protein–biased GPR3 Signaling Ameliorates Amyloid Pathology in a Preclinical Alzheimer's Disease Mouse Model,” Proceedings of the National Academy of Sciences 119 (2022): 2204828119, 10.1073/pnas.2204828119. PMC9546571 36161942

[38] M. O'Hayre , K. Eichel , S. Avino , et al., “Genetic Evidence That β‐arrestins Are Dispensable for the Initiation of β2‐adrenergic Receptor Signaling to ERK,” Science Signaling 10 (2017): aal3395. 10.1126/scisignal.aal3395 PMC5751434 28634209

[39] A. Strauss , A. J. Gonzalez‐Hernandez , J. Lee , et al., “Structural Basis of Positive Allosteric Modulation of Metabotropic Glutamate Receptor Activation and Internalization,” Nature Communications 15 (2024): 6498, 10.1038/s41467-024-50548-x. PMC11294631 39090128

[40] H. Oakley , S. L. Cole , S. Logan , et al., “Intraneuronal β‐Amyloid Aggregates, Neurodegeneration, and Neuron Loss in Transgenic Mice with Five Familial Alzheimer's Disease Mutations: Potential Factors in Amyloid Plaque Formation,” Journal of Neuroscience 26 (2006): 10129–10140, 10.1523/JNEUROSCI.1202-06.2006. 17021169 PMC6674618

[41] R. d'Isa , G. Comi , and L. Leocani , “Apparatus Design and Behavioural Testing Protocol for the Evaluation of Spatial Working Memory in Mice through the Spontaneous Alternation T‐maze,” Scientific Reports 11 (2021): 21177, 10.1038/s41598-021-00402-7. 34707108 PMC8551159

[42] E. E. Clarke and M. S. Shearman , “Quantitation of Amyloid‐β Peptides in Biological Milieu Using a Novel Homogeneous Time‐resolved Fluorescence (HTRF) Assay,” Journal of Neuroscience Methods 102 (2000): 61–68, 10.1016/S0165-0270(00)00280-6. 11000412

[43] N. Meyer , J. Bentin , J.‐M. Janot , et al., “Ultrasensitive Detection of Aβ42 Seeds in Cerebrospinal Fluid with a Nanopipette‐based Real‐time Fast Amyloid Seeding and Translocation Assay,” Analytical Chemistry 95 (2023): 12623–12630, 10.1021/acs.analchem.3c00017. 37587130

[44] G. J. Pagnier , K. V. Kastanenka , M. Sohn , et al., “Novel Botanical Drug DA‐9803 Prevents Deficits in Alzheimer's Mouse Models,” Alzheimer's Research & Therapy 10 (2018): 11, 10.1186/s13195-018-0338-2. PMC5789736 29378621

[45] M. Kurita , T. Holloway , A. García‐Bea , et al., “HDAC2 Regulates Atypical Antipsychotic Responses Through the Modulation of mGlu2 Promoter Activity,” Nature Neuroscience 15 (2012): 1245–1254, 10.1038/nn.3181. 22864611 PMC3431440

[46] J. DelaCuesta‐Barrutia , O. Martínez‐Peula , G. Rivero , et al., “Effect of Antipsychotic Drugs on Group II Metabotropic Glutamate Receptor Expression and Epigenetic Control in Postmortem Brains of Schizophrenia Subjects,” Translational Psychiatry 14 (2024): 1–10, 10.1038/s41398-024-02832-z. 38396013 PMC10891050

[47] S. Chiechio , A. Copani , M. Zammataro , G. Battaglia , R. W. G. IV , and F. Nicoletti , “Transcriptional Regulation of Type‐2 Metabotropic Glutamate Receptors: an Epigenetic Path to Novel Treatments for Chronic Pain,” Trends in Pharmacological Sciences 31 (2010): 153–160, 10.1016/j.tips.2009.12.003. 20064669

[48] D. Durand , L. Carniglia , J. Beauquis , C. Caruso , F. Saravia , and M. Lasaga , “Astroglial mGlu3 Receptors Promote Alpha‐secretase‐mediated Amyloid Precursor Protein Cleavage,” Neuropharmacology 79 (2014): 180–189, 10.1016/j.neuropharm.2013.11.015. 24291464

[49] A. Thathiah , K. Horré , A. Snellinx , et al., “β‐arrestin 2 Regulates Aβ Generation and γ‐secretase Activity in Alzheimer's Disease,” Nature Medicine 19 (2013): 43–49, 10.1038/nm.3023. 23202293

[50] J. Liu , L. Xue , M. A. Ravier , et al., “Multi‐faceted Roles of β‐arrestins in G Protein‐coupled Receptor Endocytosis,” Nature Communications 17 (2025): 463, 10.1038/s41467-025-67156-y. PMC12799616 41381542

[51] A. Thathiah and B. De Strooper , “The Role of G Protein‐coupled Receptors in the Pathology of Alzheimer's Disease,” Nature Reviews Neuroscience 12 (2011): 73–87, 10.1038/nrn2977. 21248787

[52] X. Zhang and W. Song , “The Role of APP and BACE1 Trafficking in APP Processing and Amyloid‐β Generation,” Alzheimer's Research & Therapy 5 (2013): 46, 10.1186/alzrt211. PMC3978418 24103387

[53] C. Haass , C. Kaether , G. Thinakaran , and S. Sisodia , “Trafficking and Proteolytic Processing of APP,” Cold Spring Harbor Perspectives in Medicine 2 (2012): a006270, 10.1101/cshperspect.a006270. 22553493 PMC3331683

[54] L. Teng , J. Zhao , F. Wang , L. Ma , and G. Pei , “A GPCR/Secretase Complex Regulates β‐ and γ‐secretase Specificity for Aβ Production and Contributes to AD Pathogenesis,” Cell Research 20 (2010): 138–153, 10.1038/cr.2010.3. 20066010

[55] S. Eggert , C. Thomas , S. Kins , and G. Hermey , “Trafficking in Alzheimer's Disease: Modulation of APP Transport and Processing by the Transmembrane Proteins LRP1, SorLA, SorCS1c, Sortilin, and Calsyntenin,” Molecular Neurobiology 55 (2018): 5809–5829, 10.1007/s12035-017-0806-x. 29079999

[56] G. Thinakaran and E. H. Koo , “Amyloid Precursor Protein Trafficking, Processing, and Function,” Journal of Biological Chemistry 283 (2008): 29615–29619, 10.1074/jbc.R800019200. 18650430 PMC2573065

[57] C. Nasca , B. Bigio , D. Zelli , F. Nicoletti , and B. S. McEwen , “Mind the Gap: Glucocorticoids Modulate Hippocampal Glutamate Tone Underlying Individual Differences in Stress Susceptibility,” Molecular Psychiatry 20 (2015): 755–763, 10.1038/mp.2014.96. 25178162 PMC4366364

[58] S. Chiechio , M. Zammataro , M. E. Morales , et al., “Epigenetic Modulation of mGlu2 Receptors by Histone Deacetylase Inhibitors in the Treatment of Inflammatory Pain,” Molecular Pharmacology 75 (2009): 1014–1020, 10.1124/mol.108.054346. 19255242

[59] C. Nasca , D. Xenos , Y. Barone , et al., “L‐acetylcarnitine Causes Rapid Antidepressant Effects through the Epigenetic Induction of mGlu2 Receptors,” Proceedings of the National Academy of Sciences 110 (2013): 4804–4809, 10.1073/pnas.1216100110. PMC3607061 23382250

[60] H. Ohishi , A. Neki , and N. Mizuno , “Distribution of a Metabotropic Glutamate Receptor, mGluR2, in the central Nervous System of the Rat and Mouse: an Immunohistochemical Study with a Monoclonal Antibody,” Neuroscience Research 30 (1998): 65–82, 10.1016/S0168-0102(97)00120-X. 9572581

[61] D. L. Taylor , L. T. Diemel , M. L. Cuzner , and J. M. Pocock , “Activation of Group II Metabotropic Glutamate Receptors Underlies Microglial Reactivity and Neurotoxicity Following Stimulation with Chromogranin A, a Peptide up‐Regulated in Alzheimer's Disease,” Journal of Neurochemistry 82 (2002): 1179–1191, 10.1046/j.1471-4159.2002.01062.x. 12358765

[62] D. F. Condorelli , P. Dell'Albani , M. Corsaro , et al., “Metabotropic Glutamate Receptor Expression in Cultured Rat Astrocytes and human Gliomas,” Neurochemical Research 22 (1997): 1127–1133, 10.1023/A:1027317319166. 9251103

[63] N. Matsumura , M. Takami , M. Okochi , et al., “γ‐Secretase Associated with Lipid Rafts,” Journal of Biological Chemistry 289 (2014): 5109–5121, 10.1074/jbc.M113.510131. 24375443 PMC3931069

[64] A. Thathiah , K. Spittaels , M. Hoffmann , et al., “The Orphan G Protein–Coupled Receptor 3 Modulates Amyloid‐Beta Peptide Generation in Neurons,” Science 323 (2009): 946–951, 10.1126/science.1160649. 19213921

[65] M. S. P. Wolfe , “Presenilin, γ‐Secretase, and the Search for Pathogenic Triggers of Alzheimer's Disease,” Biochemistry 64 (2025): 1662–1672, 10.1021/acs.biochem.4c00830. 39996369 PMC13137448

[66] R. Fluhrer , A. Friedlein , C. Haass , and J. Walter , “Phosphorylation of Presenilin 1 at the Caspase Recognition Site Regulates Its Proteolytic Processing and the Progression of Apoptosis,” Journal of Biological Chemistry 279 (2004): 1585–1593, 10.1074/jbc.M306653200. 14576165

[67] O. M. Grbovic , P. M. Mathews , Y. Jiang , et al., “Rab5‐stimulated Up‐regulation of the Endocytic Pathway Increases Intracellular β‐Cleaved Amyloid Precursor Protein Carboxyl‐terminal Fragment Levels and Aβ Production,” Journal of Biological Chemistry 278 (2003): 31261–31268, 10.1074/jbc.M304122200. 12761223

[68] W. Xu , F. Fang , J. Ding , and C. Wu , “Dysregulation of Rab5‐Mediated Endocytic Pathways in Alzheimer's Disease,” Traffic (Copenhagen, Denmark) 19 (2018): 253–262, 10.1111/tra.12547. 29314494 PMC5869093

[69] S. Kim , Y. Sato , P. S. Mohan , et al., “Evidence That the rab5 Effector APPL1 Mediates APP‐βCTF‐induced Dysfunction of Endosomes in Down syndrome and Alzheimer's Disease,” Molecular Psychiatry 21 (2016): 707–716, 10.1038/mp.2015.97. 26194181 PMC4721948

[70] L. E. Jin , M. Wang , V. C. Galvin , et al., “mGluR2 versus mGluR3 Metabotropic Glutamate Receptors in Primate Dorsolateral Prefrontal Cortex: Postsynaptic mGluR3 Strengthen Working Memory Networks,” Cerebral Cortex 28 (2018): 974–987, 10.1093/cercor/bhx005. 28108498 PMC5974790

[71] W. Gulisano , M. Melone , C. Ripoli , et al., “Neuromodulatory Action of Picomolar Extracellular Aβ42 Oligomers on Presynaptic and Postsynaptic Mechanisms Underlying Synaptic Function and Memory,” Journal of Neuroscience 39 (2019): 5986–6000, 10.1523/JNEUROSCI.0163-19.2019. 31127002 PMC6650983

[72] W. Gulisano , M. Melone , D. D. Li Puma , et al., “The Effect of Amyloid‐β Peptide on Synaptic Plasticity and Memory Is Influenced by Different Isoforms, Concentrations, and Aggregation Status,” Neurobiology of Aging 71 (2018): 51–60, 10.1016/j.neurobiolaging.2018.06.025. 30092511 PMC8948488

[73] S. Filser , S. V. Ovsepian , M. Masana , et al., “Pharmacological Inhibition of BACE1 Impairs Synaptic Plasticity and Cognitive Functions,” Biological Psychiatry 77 (2015): 729–739, 10.1016/j.biopsych.2014.10.013. 25599931

[74] L. Texidó , M. Martín‐Satué , E. Alberdi , C. Solsona , and C. Matute , “Amyloid β Peptide Oligomers Directly Activate NMDA Receptors,” Cell Calcium 49 (2011): 184–190, 10.1016/j.ceca.2011.02.001. 21349580

[75] G. Koch , F. Di Lorenzo , S. Bonnì , V. Ponzo , C. Caltagirone , and A. Martorana , “Impaired LTP‐ but Not LTD‐Like Cortical Plasticity in Alzheimer's Disease Patients,” Journal of Alzheimer's Disease 31 (2012): 593–599, 10.3233/JAD-2012-120532. 22647254

[76] D. S. Knopman , D. T. Jones , and M. D. Greicius , “Failure to Demonstrate Efficacy of aducanumab: an Analysis of the EMERGE and ENGAGE Trials as Reported by Biogen, December 2019,” Alzheimer's & Dementia 17 (2021): 696–701, 10.1002/alz.12213. 33135381

[77] B. De Strooper and E. Karran , “The Cellular Phase of Alzheimer's Disease,” Cell 164 (2016): 603–615, 10.1016/j.cell.2015.12.056. 26871627

[78] C. Haass and D. J. Selkoe , “Soluble Protein Oligomers in Neurodegeneration: Lessons from the Alzheimer's Amyloid β‐peptide,” Nature Reviews Molecular Cell Biology 8 (2007): 101–112, 10.1038/nrm2101. 17245412

[79] G. M. Shankar , S. Li , T. H. Mehta , et al., “Amyloid‐β Protein Dimers Isolated Directly from Alzheimer's Brains Impair Synaptic Plasticity and Memory,” Nature Medicine 14 (2008): 837–842, 10.1038/nm1782. PMC2772133 18568035

[80] S. Li , S. Hong , N. E. Shepardson , D. M. Walsh , G. M. Shankar , and D. Selkoe , “Soluble Oligomers of Amyloid β Protein Facilitate Hippocampal Long‐Term Depression by Disrupting Neuronal Glutamate Uptake,” Neuron 62 (2009): 788–801, 10.1016/j.neuron.2009.05.012. 19555648 PMC2702854

[81] C. Perruchini , F. Pecorari , J.‐P. Bourgeois , C. Duyckaerts , F. Rougeon , and P. Lafaye , “Llama VHH Antibody Fragments against GFAP: Better Diffusion in Fixed Tissues than Classical Monoclonal Antibodies,” Acta Neuropathologica 118 (2009): 685–695, 10.1007/s00401-009-0572-6. 19597828

[82] P. Debie , C. Lafont , M. Defrise , et al., “Size and Affinity Kinetics of Nanobodies Influence Targeting and Penetration of Solid Tumours,” Journal of Controlled Release 317 (2020): 34–42, 10.1016/j.jconrel.2019.11.014. 31734445

[83] G. Caljon , V. Caveliers , T. Lahoutte , et al., “Using Microdialysis to Analyse the Passage of Monovalent Nanobodies through the Blood–brain Barrier,” British Journal of Pharmacology 165 (2012): 2341–2353, 10.1111/j.1476-5381.2011.01723.x. 22013955 PMC3413867

[84] R. Faresjö , E. O. Sjöström , T. Dallas , et al., “Single Domain Antibody‐scFv Conjugate Targeting Amyloid β and TfR Penetrates the Blood–brain Barrier and Interacts with Amyloid β,” MAbs 16 (2024): 2410968, 10.1080/19420862.2024.2410968. 39358860 PMC11451328

[85] J. R. Haynes , C. A. Whitmore , W. J. Behof , et al., “Targeting Soluble Amyloid‐beta Oligomers with a Novel Nanobody,” Scientific Reports 14 (2024): 16086, 10.1038/s41598-024-66970-6. 38992064 PMC11239946

[86] P. Lafaye , I. Achour , P. England , C. Duyckaerts , and F. Rougeon , “Single‐domain Antibodies Recognize Selectively Small Oligomeric Forms of Amyloid β, Prevent Aβ‐induced Neurotoxicity and Inhibit Fibril Formation,” Molecular Immunology 46 (2009): 695–704, 10.1016/j.molimm.2008.09.008. 18930548

[87] C. Danis , E. Dupré , T. Bouillet , et al., “Inhibition of Tau Neuronal Internalization Using Anti‐tau Single Domain Antibodies,” Nature Communications 16 (2025): 3162, 10.1038/s41467-025-58383-4. PMC11965310 40175345

[88] J. Benn , S. Cheng , S. Keeling , et al., “Aggregate‐selective Removal of Pathological Tau by Clustering‐activated Degraders,” Science 385 (2024): 1009–1016, 10.1126/science.adp5186. 39208111 PMC7616837

[89] L. V. C. Miller , G. Papa , M. Vaysburd , et al., “Co‐opting Templated Aggregation to Degrade Pathogenic Tau Assemblies and Improve Motor Function,” Cell 187 (2024): 5967–5980.e17, 10.1016/j.cell.2024.08.024. 39276772 PMC7616835

[90] T. Li , M. Vandesquille , F. Koukouli , et al., “Camelid Single‐domain Antibodies: a Versatile Tool for in vivo Imaging of Extracellular and Intracellular Brain Targets,” Journal of Controlled Release 243 (2016): 1–10, 10.1016/j.jconrel.2016.09.019. 27671875

[91] R. M. Deacon , “Assessing Nest Building in Mice,” Nature Protocols 1 (2006): 1117–1119, 10.1038/nprot.2006.170. 17406392

[92] C. Samaey , A. Schreurs , S. Stroobants , and D. Balschun , “Early Cognitive and Behavioral Deficits in Mouse Models for Tauopathy and Alzheimer's Disease,” Frontiers in Aging Neuroscience 11 (2019): 335, 10.3389/fnagi.2019.00335. 31866856 PMC6908963

[93] P. Giannoni , F. Gaven , D. de Bundel , et al., “Early Administration of RS 67333, a Specific 5‐HT4 Receptor Agonist, Prevents Amyloidogenesis and Behavioral Deficits in the 5XFAD Mouse Model of Alzheimer's Disease,” Frontiers in Aging Neuroscience 5 (2013): 96, 10.3389/fnagi.2013.00096. 24399967 PMC3871961

[94] P.‐A. Lafon , Y. Wang , M. Arango‐Lievano , et al., “Fungicide Residues Exposure and β‐amyloid Aggregation in a Mouse Model of Alzheimer's Disease,” Environmental Health Perspectives 128 (2020): 17011, 10.1289/EHP5550. 31939705 PMC7015540

[95] C. Ruehl‐Fehlert , B. Kittel , G. Morawietz , et al., “Revised Guides for Organ Sampling and Trimming in Rats and Mice—Part 1,” Experimental and Toxicologic Pathology 55 (2003): 91–106, 10.1078/0940-2993-00311. 14620530

[96] B. Kittel , C. Ruehl‐Fehlert , G. Morawietz , et al., “Revised Guides for Organ Sampling and Trimming in Rats and Mice—Part 2,” Experimental and Toxicologic Pathology 55 (2004): 413–431, 10.1078/0940-2993-00349. 15384248

[97] G. Morawietz , C. Ruehl‐Fehlert , B. Kittel , et al., “Revised Guides for Organ Sampling and Trimming in Rats and Mice—Part 3,” Experimental and Toxicologic Pathology 55 (2004): 433–449, 10.1078/0940-2993-00350. 15384249

[98] W. Thong‐Asa and K. Tilokskulchai , “Neuronal Damage of the Dorsal Hippocampus Induced by Long‐term Right Common Carotid Artery Occlusion in Rats,” Iranian Journal of Basic Medical Sciences 17 (2014): 220–226. 24847426 PMC4016694

