# Supplementary material for: Divergent Roles of mGlu2 and mGlu3 Receptors in Amyloid‐β Production and Cognitive Dysfunctions in Alzheimer's Disease
Source: Adv Sci (Weinh). 2026 Apr 14:e23791. Online ahead of print. doi: 10.1002/advs.202523791 (PMC13334641; doi:10.1002/advs.202523791)
Supplement: Supplementary file 1 — Supporting File: advs75218‐sup‐0001‐SuppMat.docx. [file ADVS-9999-e23791-s001.docx]

**Supporting information**

**Divergent roles of mGlu2 and mGlu3 receptors in amyloid-β production and cognitive dysfunctions in Alzheimer's disease**

*Pierre-André Lafon^1,2*^, Mireille Elodie Tsitokana^2^†, Ugo Guy Alenda^2,3^†, Yen-Ling Lian^2^, Clémentine Eva Philibert^2^, Mathieu Oosterlaken^2^, Marta Cimadevila^2^, Gaëlle Dudon^3^, Jessica Monnic^2^, Salomé Roux^2^, Julie Bessié^2^, Séverine Diem^2^, Franck Vandermoere^2^, Laurent Prézeau^2^, Patrick Chames^4^, Julie Kniazeff^2^, Sylvie Claeysen^2^, Anaïs Menny^2^, Jean-Philippe Pin^2^, Véronique Perrier^3*^, Jianfeng Liu^1*^ and Philippe Rondard^2*^.*

^1^ Cellular Signaling Laboratory, International Research Center for Sensory Biology and Technology of MOST, Key Laboratory of Molecular Biophysics of MOE, and College of Life Science and Technology, Huazhong University of Science and Technology, 430074 Wuhan, China.

^2^ Institut de Génomique Fonctionnelle (IGF), University of Montpellier, CNRS, INSERM, 34094 Montpellier Cedex 5, France.

^3^ Institut des Neurosciences de Montpellier (INM), University of Montpellier, INSERM, CNRS, Montpellier, France.

^4^ Aix Marseille University, CNRS, INSERM, Institut Paoli-Calmettes, CRCM, Marseille, France.

***Corresponding authors:** Lafon Pierre-André: [pierre-andre.lafon@igf.cnrs.fr](mailto:pierre-andre.lafon@igf.cnrs.fr)

Perrier Véronique: [veronique.perrier@umontpellier.fr](mailto:veronique.perrier@umontpellier.fr)

Liu Jianfeng: [jfliu@mail.hust.edu.cn](mailto:jfliu@mail.hust.edu.cn)

Rondard Philippe: [philippe.rondard@igf.cnrs.fr](mailto:philippe.rondard@igf.cnrs.fr)

**This file includes:**

Figures S1 to S16

Tables S1


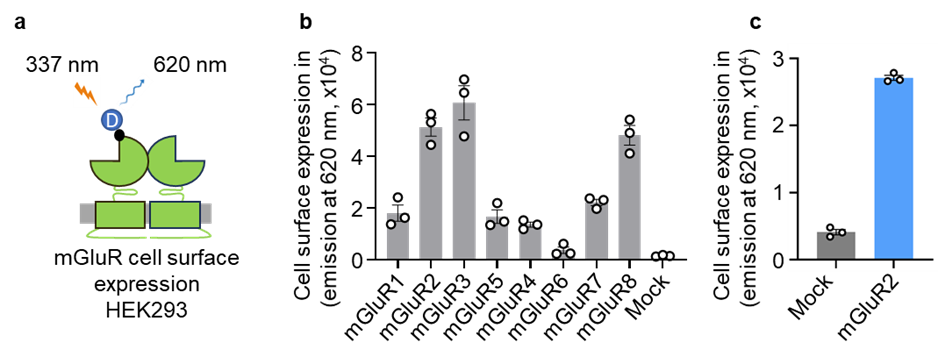


**Figure S1. Cell surface expression of transfected receptors.** (a) The relative expression of transfected Snap-tagged mGluRs (^ST^mGluRs) at the cell surface of HEK293T cells was measured using Snap-Lumi4-terbium (Tb). Expression was measured using the Tb emission at 620 nm, following excitation at 337 nm. (b) Cell surface expression levels of ^ST^mGluR1 to ^ST^mGluR8 used for the DN13-DN1 nanobody selectivity experiments. Data are presented as mean ± s.e.m. from three biologically independent experiments performed in triplicate. (c) Cell surface expression levels of ^ST^mGluR2 used in IP_1_ accumulation assays with DN13-DN1 and DN1 nanobodies. Data are presented as mean ± s.e.m. from three biologically independent experiments, each performed in triplicate.

**
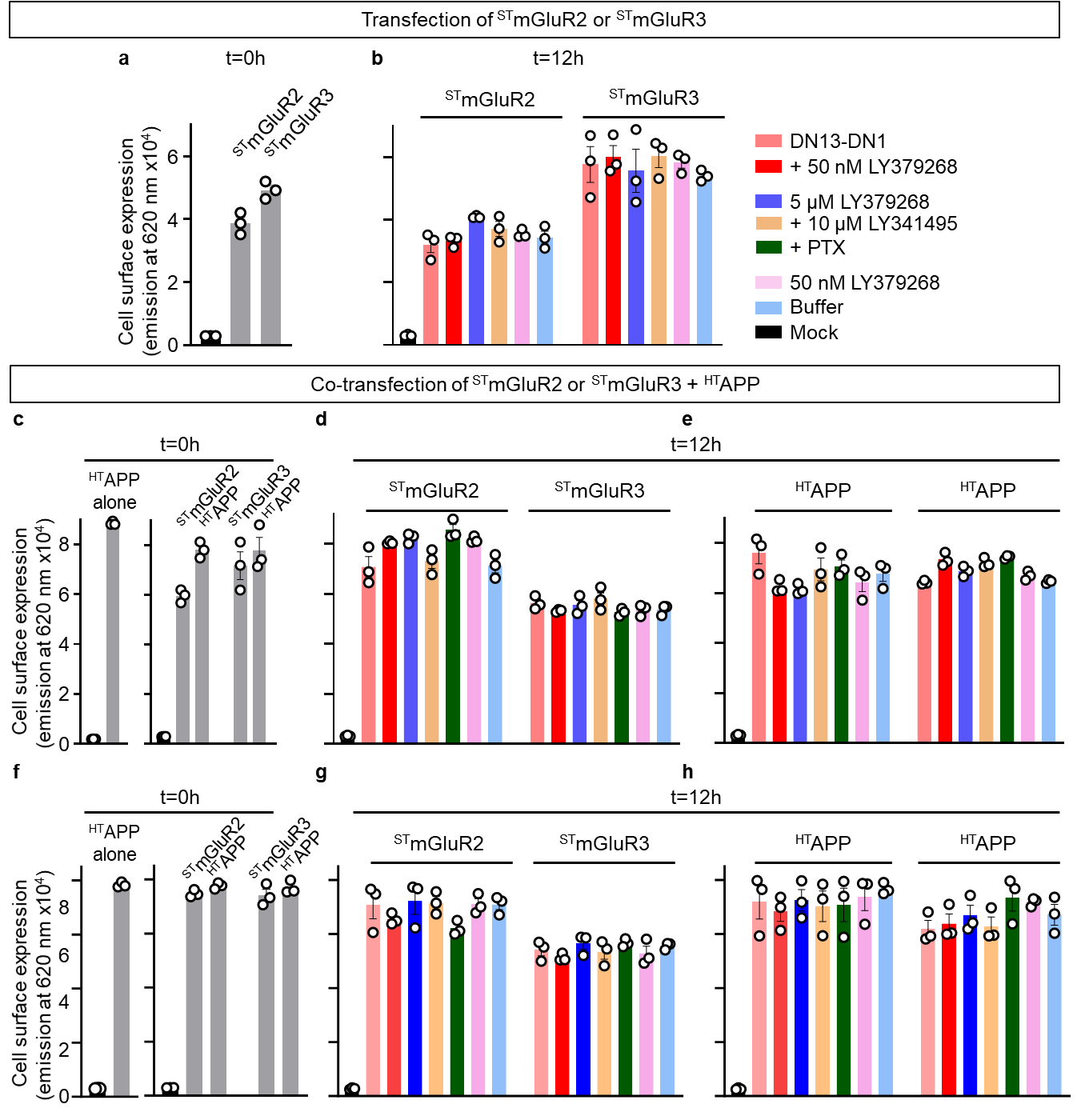
**

**Figure S2. Cell surface expression of transfected receptors.** (a,b) Quantification of the cell surface expression of Snap-tagged mGluR2 (^ST^mGluR2) or mGluR3 (^ST^mGluR3) transfected in HEK293T cells at baseline (*a*, t=0h) and following 12 hours of treatment (*b*, t=12h). Cells were treated with PBS; LY379268 (5 μM) alone; LY379268 (5 μM) following pretreatment with LY341495 (10 µM), or with pertussis toxin (PTX, 0.2 µg/mL); DN13-DN1 (200 nM) alone; LY379268 at EC_20_ (0.5 nM) alone, or the combination of DN13-DN1 (200 nM) with LY379268 EC_20_. Surface expression was assessed using Snap-Lumi4-terbium (Tb) and its emission was recorded at 620 nm upon excitation at 337 nm. (c-h) Expression of ^ST^mGluR2 or ^ST^mGluR3 co-transfected with Halo-tagged APP (^HT^APP) in HEK293T cells prior to treatment (*c*, *f*; t=0 h) or after 12 h of treatment with the different compounds described above (*d-e, g-h*). Data in *a-b* represent the expression in the conditions shown in Figure 1c, data in *d-e* correspond to Figure 1d, while data in *g-h* are the expression represented in Figure 1f-g. Data are presented as mean ± s.e.m. from three biologically independent experiments, each performed in triplicate.


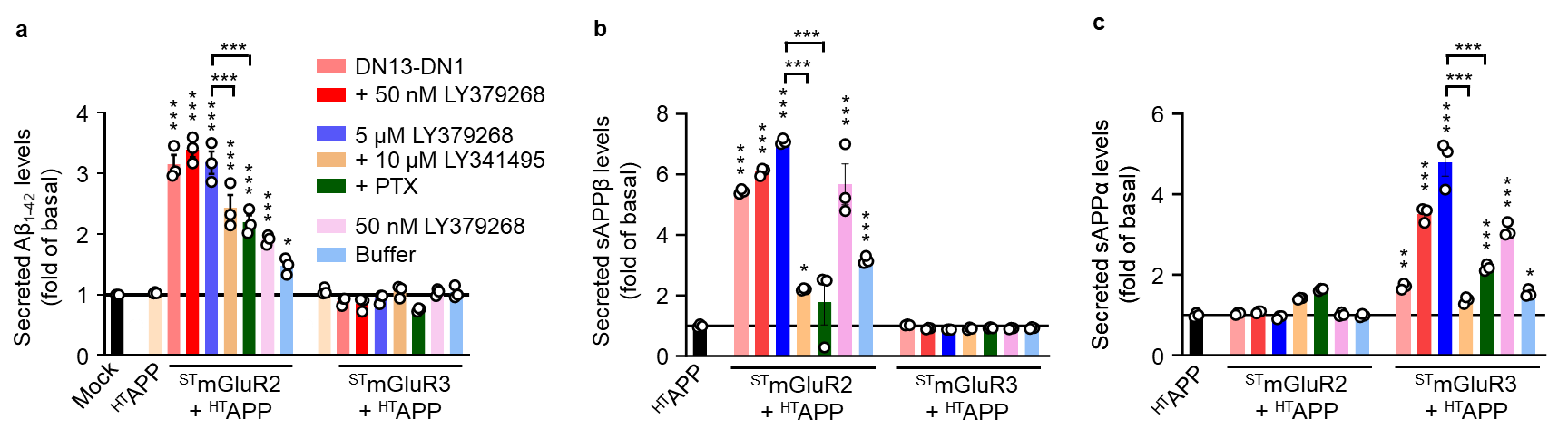


**Figure S3. Pertussis toxin inhibits mGluR2-induced Aβ_1-42_ and sAPPβ and mGluR3-induced sAPPα secretion in HEK293 cells.** Secreted Aβ_1-42_ (a), sAPPβ (b) and sAPPα (c) levels in HEK293T cells co-expressing Halo-tagged APP (^HT^APP) and either Snap-tagged mGluR2 (^ST^mGluR2) or mGluR3 (^ST^mGluR3). Cells were treated with PBS; LY379268 (5 µM) alone, LY379268 (5 µM) following pretreatment with LY341495 (10 µM), or with pertussis toxin (PTX, 0.2 µg/mL); DN13-DN1 (200 nM); or LY379268 at EC_20_ (0.5 nM) alone or in combination with DN13-DN1. Control conditions included cells transfected with empty vector (*a*, Mock) or ^HT^APP alone (*a-c*). Data are presented as mean ± s.e.m. from three biologically independent experiments, each performed in triplicate and are shown as a ratio relative to basal levels. Statistical analysis was performed using the one-way ANOVA followed by a Holm-Šidák *post-hoc* analysis (* *p* < 0.05, ** *p* < 0.01, *** *p* < 0.001).


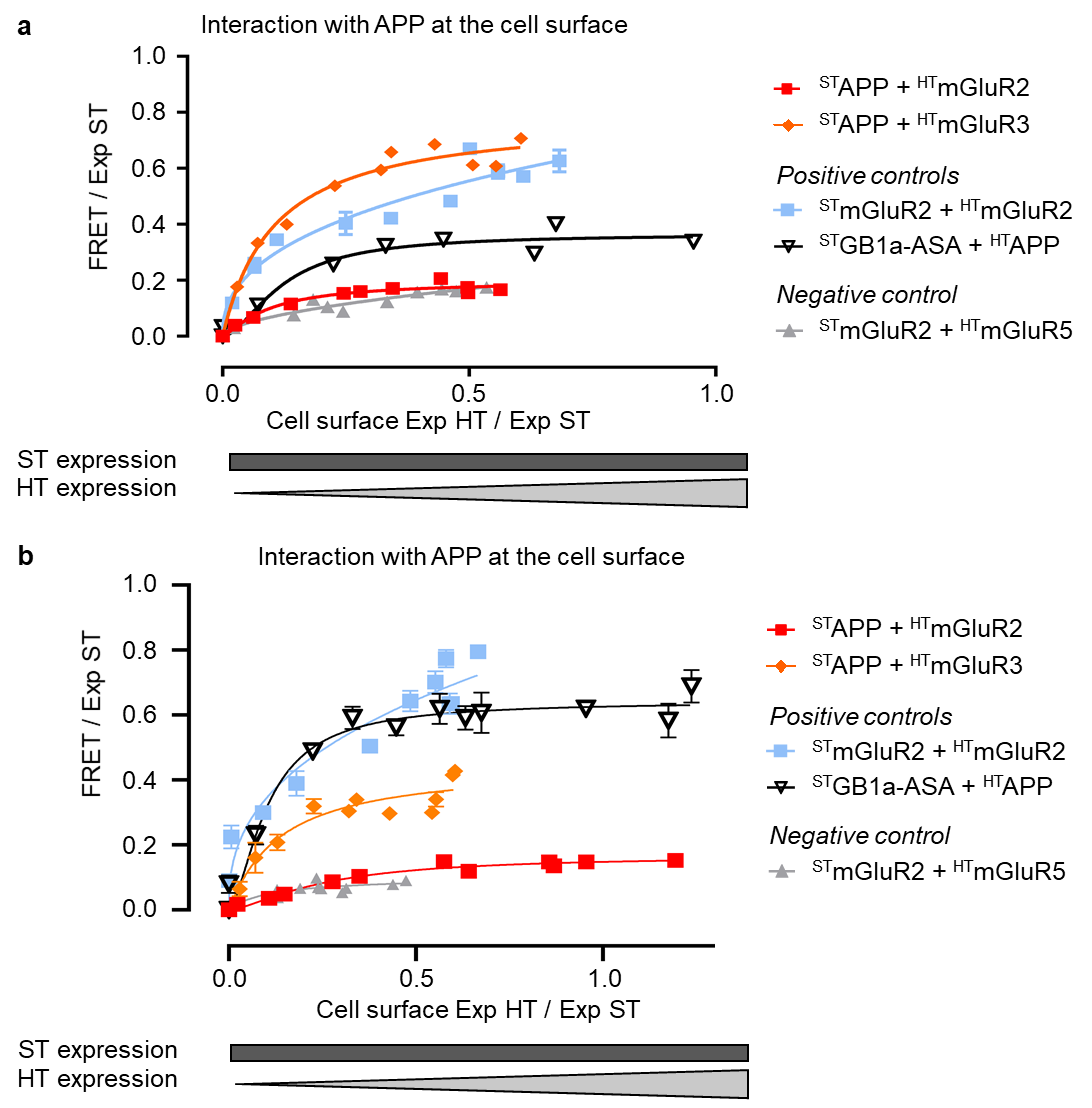


**Figure S4. Additional independent experiments of TR-FRET saturation analyses corresponding to Figure 2b.** TR-FRET saturation analyses in HEK293T cells co-expressing Snap-tagged APP (^ST^APP) with Halo-tagged mGluR3 (^HT^mGluR3; orange) or ^HT^mGluR2 (red); ^ST^mGluR2 with ^HT^mGluR2 (blue, positive control) or with ^HT^mGluR5 (grey, negative control); and ^ST^GB1a-ASA, a construct known to reach constitutively the cell surface, with ^HT^APP (black, positive control). Snap-tagged constructs were transfected at a fix cDNA amount and labelled with the donor (Snap-Lumi4-Tb labeling reagent), while increasing amounts of Halo-tagged receptors were labelled with the acceptor (Halo-red labeling reagent). Expression of cell surface Snap-tagged (Exp ST) and Halo-tagged (Exp HT) receptors as well as the FRET signal were measured. FRET/Exp ST is plotted as a function of the Halo to Snap expression ratio (Exp HT/Exp ST). Data are presented as mean ± s.e.m. of triplicate. Experiment 2 and 3 are shown in a and b, respectively.


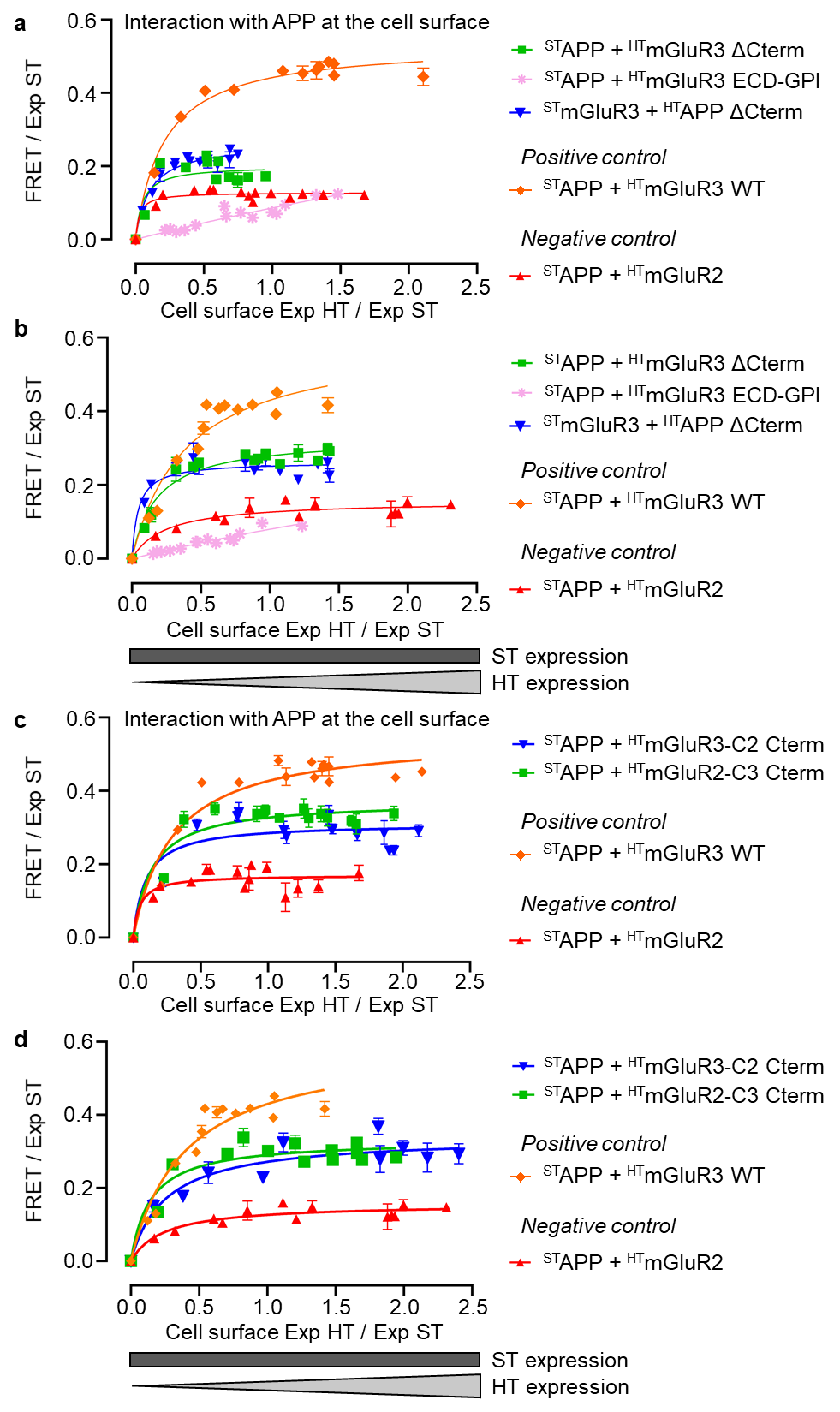


**Figure S5. Additional independent experiments of TR-FRET saturation analyses corresponding to Figure 2d and Figure 2f.** (a-b) TR-FRET saturation analyses in HEK293T cells co-expressing ^ST^APP with either ^HT^mGluR3 ECD-GPI (Del570-879; pink), a construct where the transmembrane and C-terminal regions were replaced with a GPI anchor; a C-terminally truncated variant ^HT^mGluR3-ΔCterm (Del829-879; green), ^HT^mGluR2 (red, negative control), or with ^HT^mGluR3 (orange, positive control). Additional TR-FRET experiments were conducted with ^ST^mGluR3 co-expressed with a C-terminally truncated ^HT^APP (^HT^APP-ΔCterm, Del648–695; blue). Data are presented as mean ± s.e.m. of triplicate. Experiment 2 and 3 are shown in *a* and *b*, respectively. (c-d) TR-FRET saturation analyses in HEK293T cells co-expressing ^ST^APP with ^HT^mGluR3 chimera (^HT^mGluR3-C2; blue), ^HT^mGluR2 chimera (^HT^mGluR2-C3; green), ^HT^mGluR2 (red, negative control), or ^HT^mGluR3 (orange, positive control). Data are presented as mean ± s.e.m. of triplicate. Experiment 2 and 3 are shown in *c* and *d*, respectively.


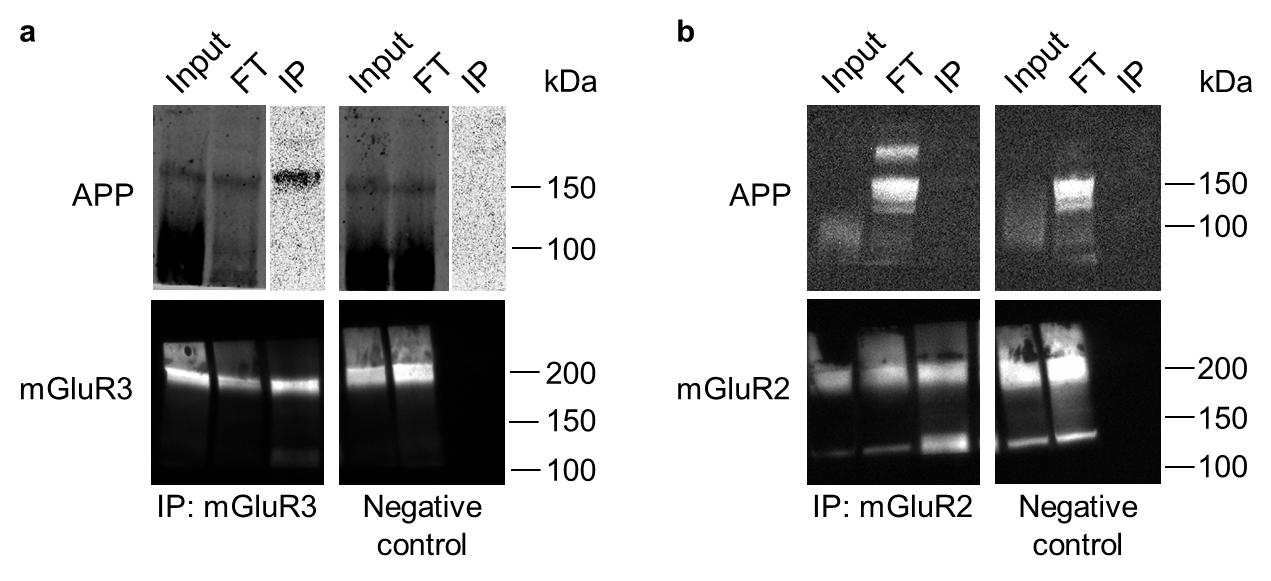


**Figure S6. mGluR3 interacts with APP, but not mGluR2, in cortical native tissues.** Representative western blots of immunoprecipitates obtained with a His-tagged silent allosteric modulator nanobody anti-mGluR3 (a) or with the His-tagged DN13-DN1 nanobody anti-mGluR2 (b) from WT mice cortex protein extracts. Inputs (cortex homogenate), flowthrough (FT) and immunoprecipitates (IP) were probed with an anti-APP antibody, anti-mGluR3 antibody and anti-mGluR2 antibody. APP was detected upon immunoprecipitation of mGluR3 but not of mGluR2. Cortex homogenates with NTA-beads and without mGluR3 or mGluR2 nanobodies were used as negative controls.


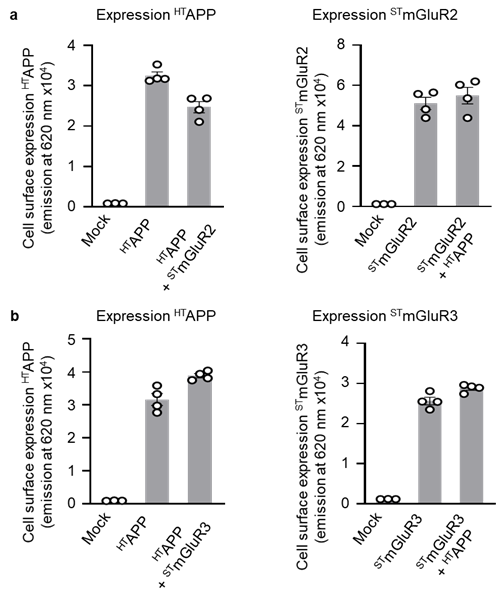


**Figure S7. Cell surface expression of transfected receptors for DERET internalization assay.** Cell surface expression of Halo-tagged APP (^HT^APP) and Snap-tagged mGluR2 (^ST^mGluR2) (a), as well as of Snap-tagged mGluR3 (^ST^mGluR3) and ^HT^APP (b), either expressed individually or co-transfected in HEK293T cells. ^ST^mGluR2 and ^ST^mGluR3 were labelled with Snap-Lumi4-terbium (Tb) labeling reagent, and ^HT^APP was labelled with Halo-Lumi4-Tb labeling reagent. Tb emission was recorded at 620 nm upon excitation at 337 nm. Expression levels in *a* correspond to the conditions shown in Figure 3b-c, and those in *b* to Figure 4d-e. Data are presented as mean ± s.e.m. from three biologically independent experiments, each performed in triplicate.


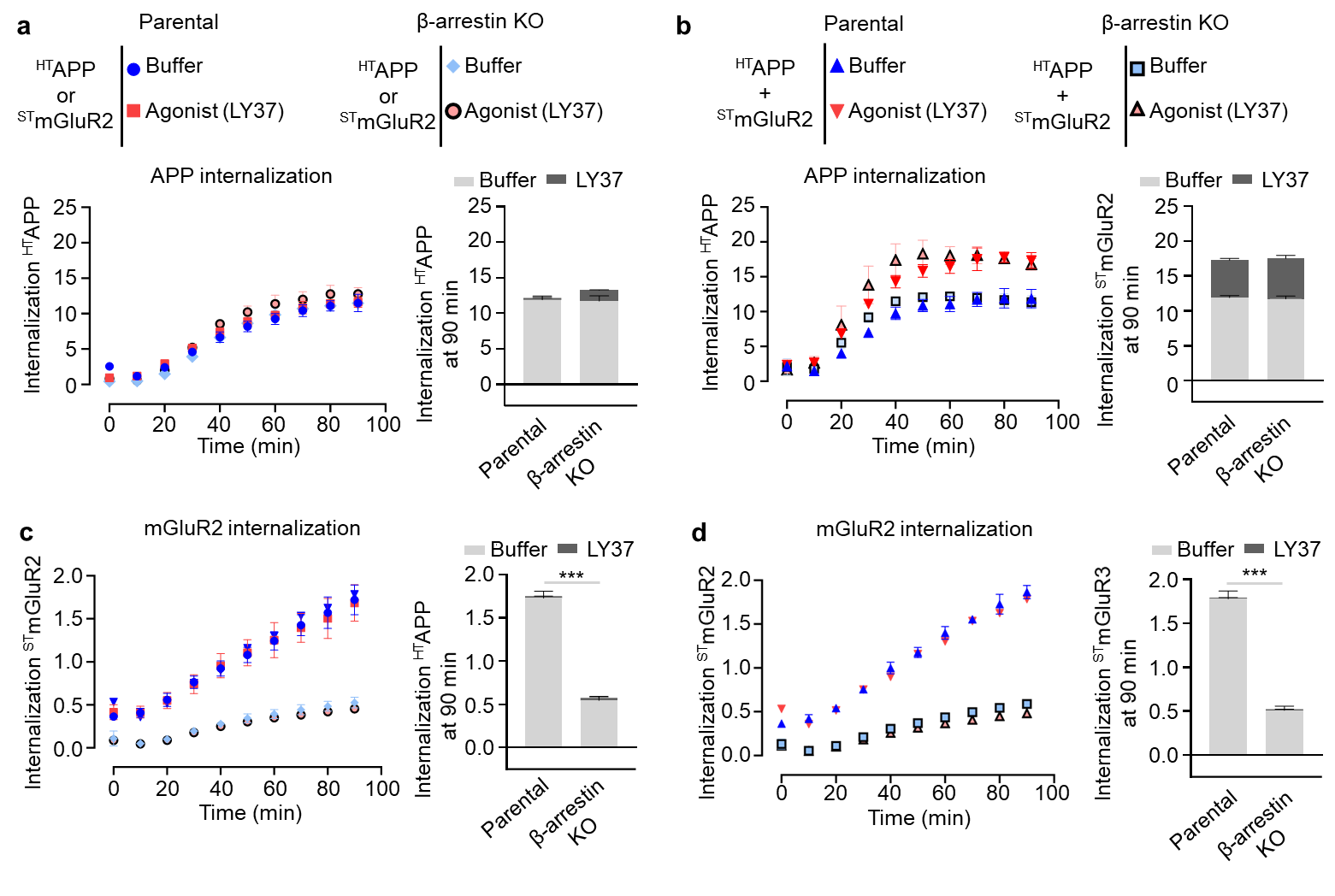


**Figure S8. APP internalization upon mGluR2 activation is β-arrestin independent.** Kinetics of internalization of Halo-tagged APP (^HT^APP) and Snap-tagged mGluR2 (^ST^mGluR2) in HEK293T cells, transfected individually (a,c) or together (b,d). Cells were treated with buffer (PBS) or with 1 µM of LY379268 (LY37). Bars represent constitutive internalization (light grey, basal signal) and agonist induced internalization (dark grey, calculated by subtracting basal signal), measured at 90 min of internalization. Data are presented as mean ± s.e.m. from three biologically independent experiments, performed in triplicate. Statistical analysis was performed using Welch’s t test (*** *p* < 0.001).


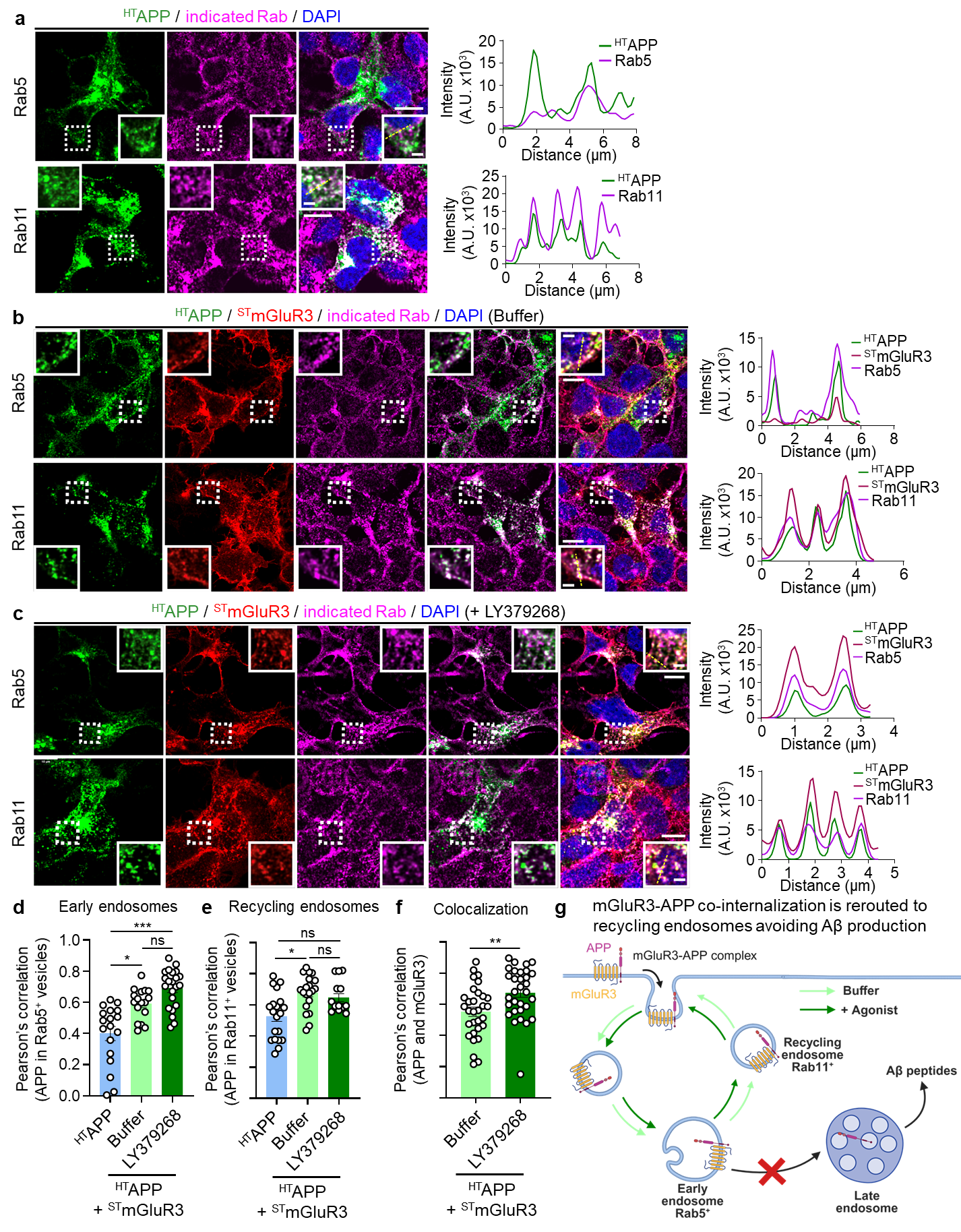


**Figure S9. Subcellular localization of the mGluR3-APP complex in Rab5^+^ and Rab11^+^ endosomal compartments in HEK cells.** (a-c) Representative confocal images of HEK293T transfected with Halo-tagged APP (^HT^APP, green) alone (*a*) or co-transfected with Snap-tagged mGluR3 (^ST^mGluR3, red, *b-c*) treated with buffer or 5 µM of LY379268. Early endosomes and recycling vesicles were identified using anti-Rab5 and anti-Rab11 antibodies (magenta), respectively. Yellow lines in the merged images denote the regions used for the corresponding fluorescence intensity histograms, showing the overlap of APP, mGluR3, and Rab signals. Images are representative of more than 20 cells. Scale bars: 10 μm and 2 μm (enlarged images). (d-f) Pearson’s correlation coefficient analysis quantifying the colocalization of APP within Rab5^+^ (*d*) and Rab11^+^ (*e*) vesicles, as well as the colocalization of APP with mGluR3 (*f*) within these compartments. Data are presented as mean ± s.e.m. Statistical analysis was performed using the Kruskal-Wallis test followed by a Dunn’s *post-hoc* analysis (*d-e*, * *p* < 0.05, *** *p* < 0.001) or Mann-Whitney tests (*f*, * *p* < 0.05). (g) Proposed model of mGluR3-APP endosomal trafficking. With or without ligand, part of the mGluR3-APP complexes is internalized into Rab5^+^ early endosomes and rerouted into Rab11^+^ recycling endosomes. Created with Biorender.com.


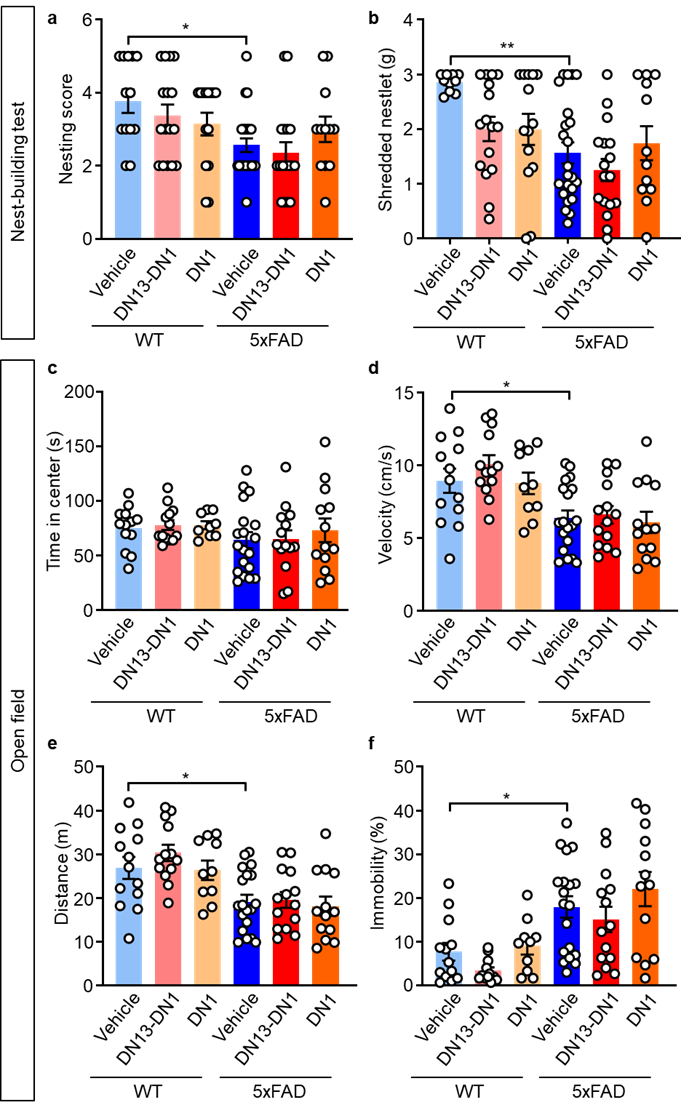


**Figure S10. Treatment with nanobodies does not affect nesting behavior or locomotor activity in 5xFAD and WT animals.** (a-b) Nesting score (*a*) and amount of shredded nestlet (*b*) in the nest-building test for WT and 5xFAD mice treated with vehicle, 10 mg/kg of DN13-DN1, or with 10 mg/kg of DN1 nanobodies. (c-f) Velocity (*c*), time spent in the center of the arena (*d*), total distance travelled (*e*), and percentage of immobility (*f*) in the arena during the habituation phase of the novel object recognition test for WT and 5xFAD mice treated with vehicle, DN13-DN1, or with DN1 nanobodies. All data are presented as mean ± s.e.m. (n = 12-21 mice / group) and analyzed using the two-way ANOVA followed by a Holm-Šidák *post-hoc* analysis (* *p* < 0.05, ** *p* < 0.01, *** *p* < 0.001).


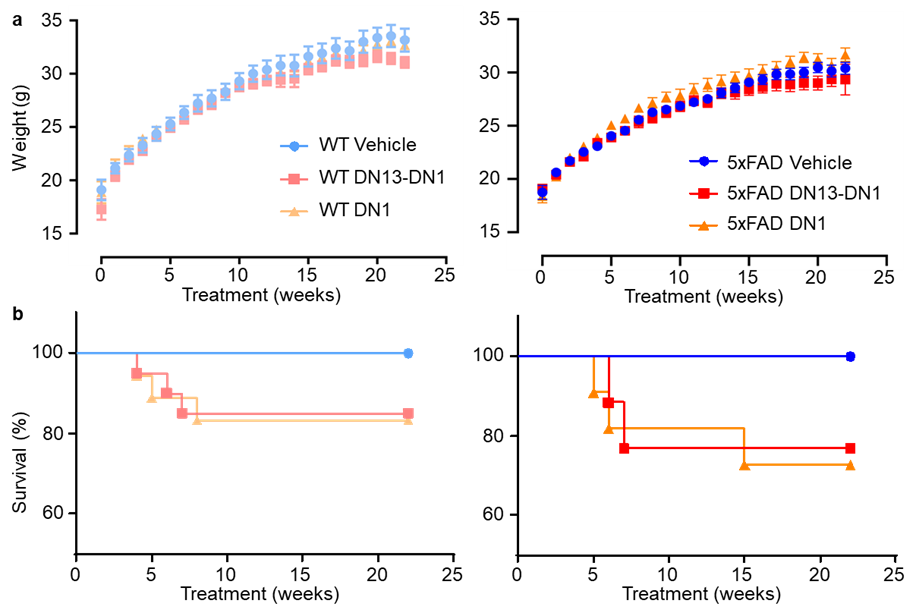


**Figure S11. Weight and survival of WT and 5xFAD mice.** (a) Body weight evolution of WT and 5xFAD mice treated weekly for 22 weeks with 10 mg/kg DN13-DN1, 10 mg/kg DN1, or vehicle. (b) Kaplan–Meier survival curves for WT and 5xFAD mice across the same treatment groups. Data are presented as mean ± s.e.m. (*a*) or percentage of survival (*b*).


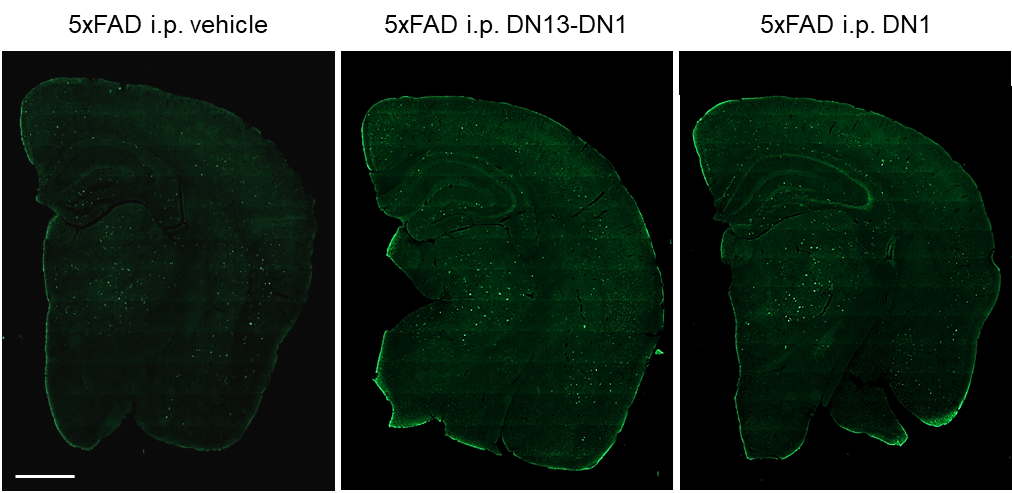


**Figure S12. Representative images of amyloid plaque burden in 5xFAD hemibrains following chronic nanobody treatment.** Thioflavin T (ThT)-stained coronal brain sections from 26-week-old 5xFAD mice chronically treated with 10 mg/kg DN13-DN1, 10 mg/kg DN1, or vehicle. Mosaic images were acquired using a slide scanner Axio Scan.Z1 with a 20× objective. Scale bar, 1 mm.


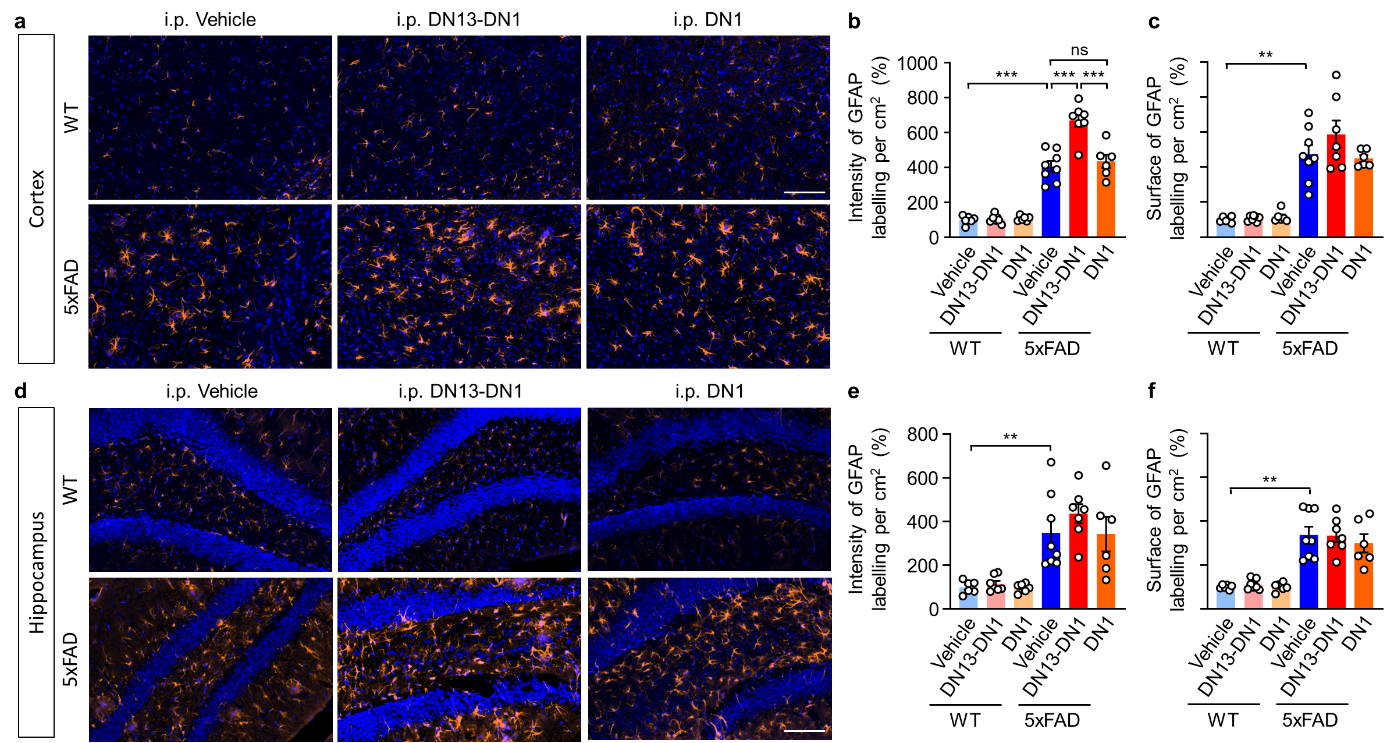


**Figure S13. DN13-DN1 nanobody increases astrogliosis in 5xFAD mice.** (a) Representative images of GFAP labelling showing astrocytes in the cortex of 26-week-old WT and 5xFAD mice chronically treated with 10 mg/kg (for 22 weeks, once a week) of either DN13-DN1, DN1 or with vehicle. Whole-section mosaic images were acquired using a slide scanner Axio Scan.Z1 with a 20x objective (scale bar: 100 µm). (b-c) Quantification of the intensity (*b*) and surface area (*c*) of the GFAP labelling normalized by the total cortical area analyzed in WT and 5xFAD mice. *d*) Representative GFAP-labelled astrocytes in the hippocampus of WT and 5xFAD mice for the same treatment groups. Images were acquired as mosaics using a 20x objective (scale bar: 100 µm). (e-f) Quantification of the intensity (*e*) and surface (*h*) of labelling normalized by the hippocampal area in WT and 5xFAD mice. Data in panels *b-c* and *e-f* are presented as mean ± s.e.m., normalized to WT vehicle (%) (n = 6-8 animals / group, 13-23 sections / animal) and analyzed using the two-way ANOVA followed by a Holm-Šidák *post-hoc* analysis (** *p* < 0.01; *** *p* < 0.001).


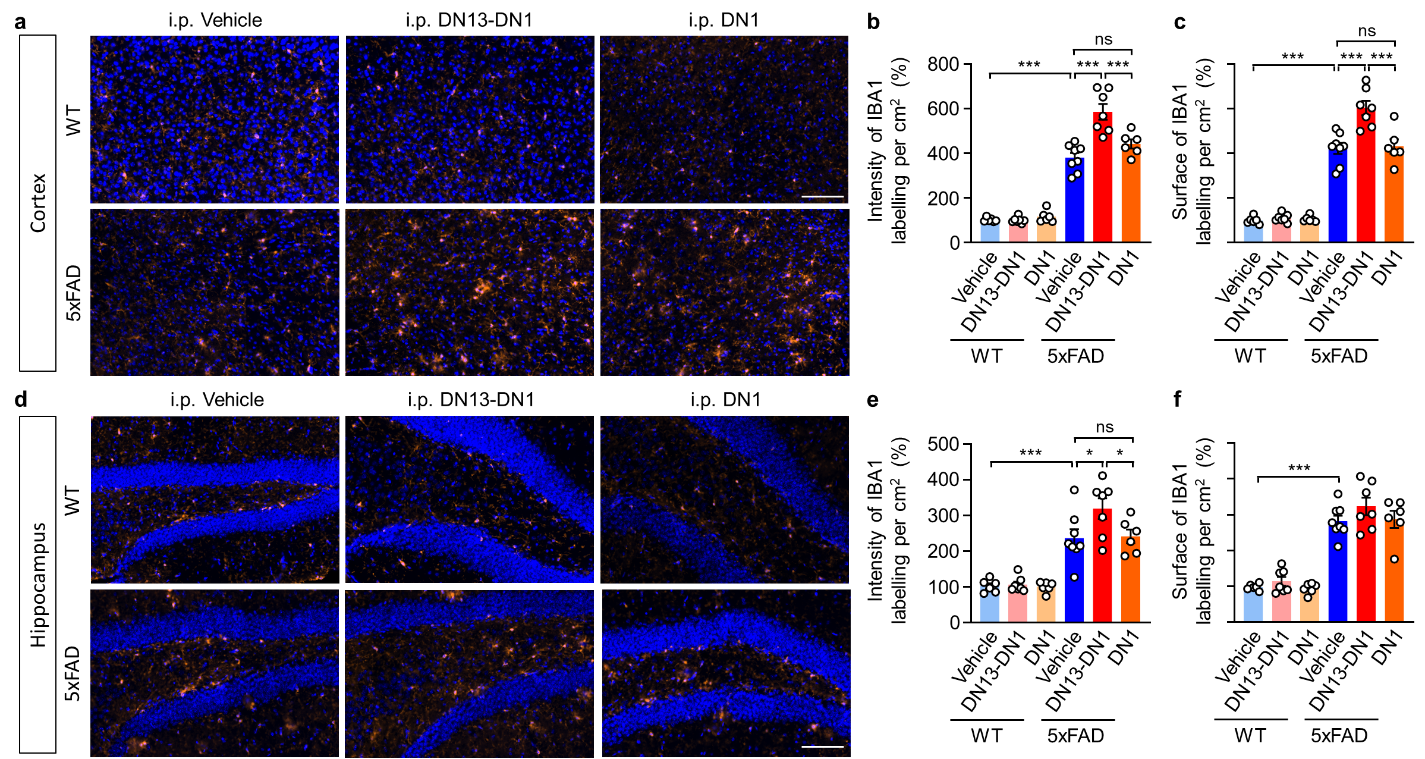


**Figure S14. DN13-DN1 nanobody increases microglial reactivity in 5xFAD mice.** (a) Representative images of IBA1 labelling showing microglial cells in the cortex of 26-week-old WT and 5xFAD mice chronically treated with 10 mg/kg (for 22 weeks, once a week) of either DN13-DN1, DN1 or with vehicle. Whole-section mosaic images were acquired using a slide scanner Axio Scan.Z1 with a 20x objective (scale bar: 100 µm). (b-c) Quantification of the intensity *(b)* and surface area *(c)* of the IBA1 labelling normalized by the cortical area analyzed in WT and 5xFAD mice. (d) Representative IBA1-labelled astrocytes in the hippocampus of WT and 5xFAD mice for the same treatment groups. Images were acquired as mosaics using a 20x objective (scale bar: 100 µm). (e-f) Quantification of the intensity *(e)* and surface *(f)* of labelling normalized by the hippocampal area in WT and 5xFAD mice. Data in panels *b-c* and *e-f* are presented as mean ± s.e.m., normalized to WT vehicle (%) (n = 6-8 animals / group, 13-23 sections / animal) and analyzed using the two-way ANOVA followed by a Holm-Šidák *post-hoc* analysis (** *p* < 0.01; *** *p* < 0.001).

**
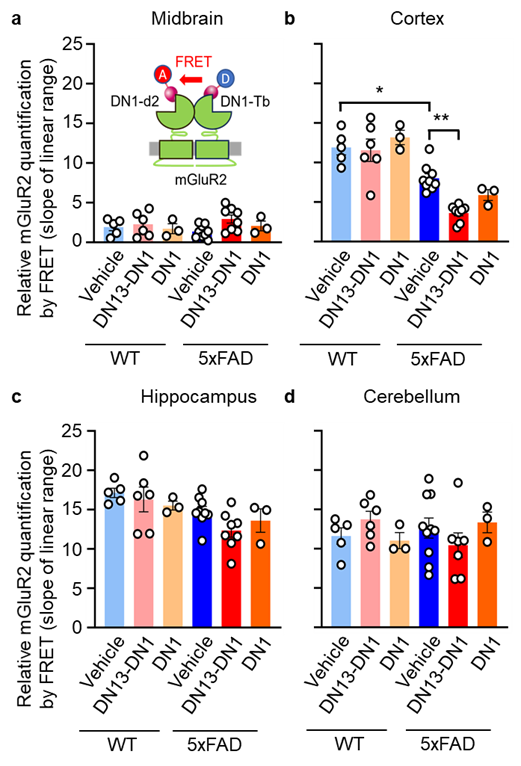
**

**Figure S15. DN13-DN1 nanobody treatment reduces mGluR2 homodimers levels in the cortex of 5xFAD mice.** (a) Schematic representation of the TR-FRET-based assay used for the relative quantification of mGluR2 homodimers, using DN1 nanobodies conjugated to a donor (DN1-Tb) and an acceptor (DN1-d2), each at 25 nM. (a-d) Relative quantification of mGluR2 homodimers in the midbrain (*a*), cortex (*b*), hippocampus (*c*), and cerebellum (*d*) of WT and 5xFAD mice chronically treated with 10 mg/kg of DN13-DN1, DN1, or with vehicle. The TR-FRET signal corresponds to the slope values from relative linear quantification curves. Data are presented as mean ± s.e.m. (n = 3-9 animals / group) and were analyzed using the two-way ANOVA followed by a Holm-Šidák *post hoc* analysis (* *p* < 0.05, ** *p* < 0.01).


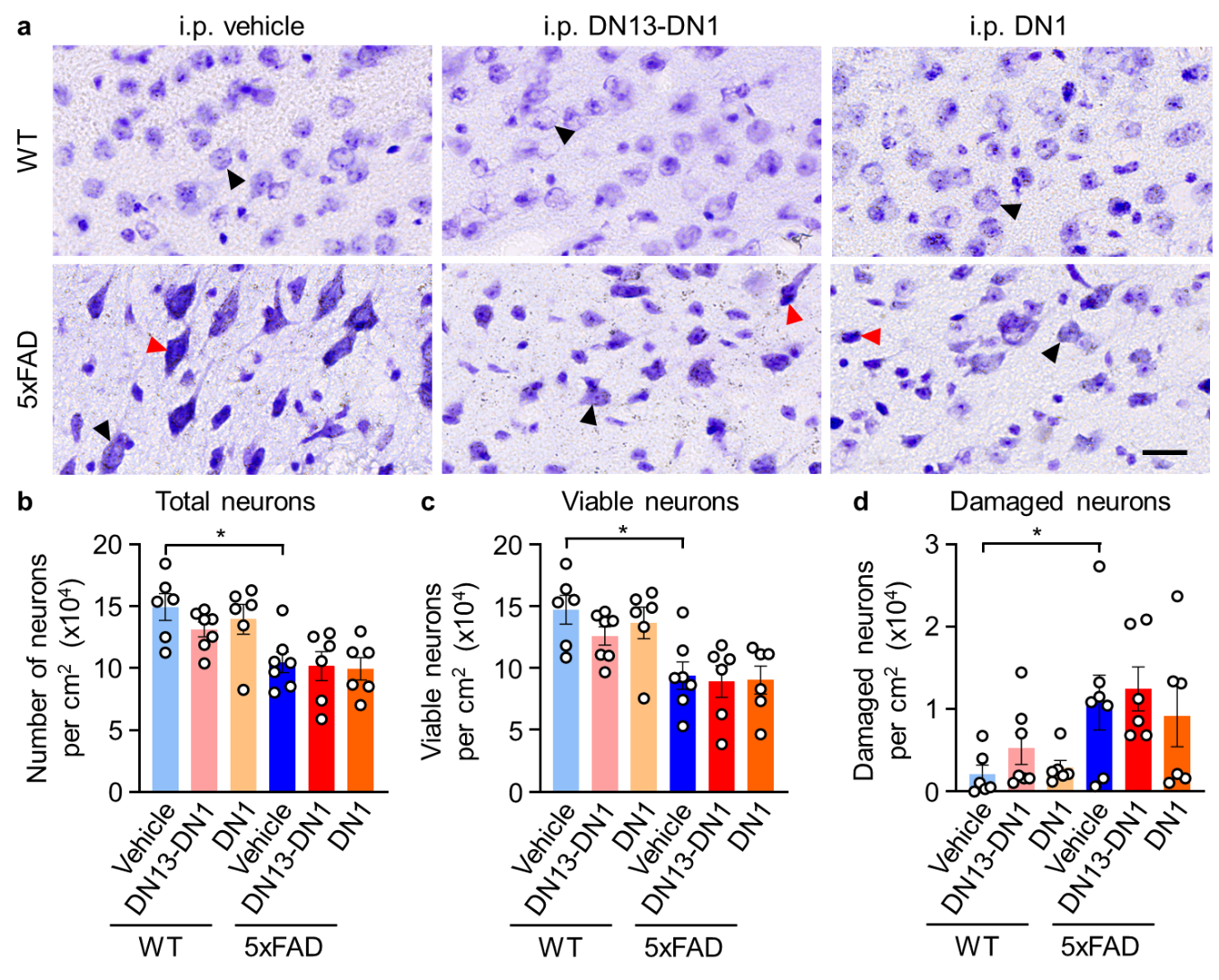


**Figure S16. DN13-DN1 nanobody treatment does not induce neuronal loss in the cortex of 5xFAD mice.** (a) Representative images of cresyl violet staining showing neurons in the cortex of 26-week-old WT and 5xFAD mice chronically treated with 10 mg/kg of either DN13-DN1, DN1 or with vehicle. Black and red arrowheads highlight viable and damaged neurons, respectively. Whole-section mosaic images were acquired using a slide scanner Axio Scan.Z1 with a 20x objective (scale bar: 20 µm). (b-d) Quantification of the total number of cortical neurons (*b*), then classified into viable (*c*) and damaged (*d*) neurons normalized by the total cortex area analyzed in WT and 5xFAD mice. Data in panels are presented as mean ± s.e.m. (n = 6-7 animals / group, 4-6 sections / animal) and analyzed using the two-way ANOVA followed by a Holm-Šidák post hoc analysis (* *p* < 0.05).

| **Organ** | **WT i.p. vehicle** | **WT i.p. DN1** | **WT i.p. DN13-DN1** |
| --- | --- | --- | --- |
| Adipose tissue | WNL | WNL + mononuclear cell infiltrate (slight multifocal) | WNL + mononuclear cell infiltrate (marked, diffuse) |
| Adrenal gland | WNL | WNL | Presence of apparent multinucleate giant cells within the adrenal medulla |
| Aorta | WNL | WNL | WNL + vasculitis in adjacent blood vessels |
| Bladder | WNL | WNL | WNL |
| Caecum | WNL | WNL | WNL |
| Epididymis | WNL | WNL | WNL |
| Esophagus | WNL | WNL | WNL |
| Eyes with sciatic nerve | WNL | WNL | WNL |
| Harderian gland | WNL | WNL | WNL |
| Heart and aorta | WNL | WNL | WNL |
| Intestines (roll, duodenum, ileum, jejunum and colon) | WNL | WNL | WNL |
| Kidney | WNL | Cortical proximal tubular basophilia (diffuse, moderate) | Cortical proximal tubular basophilia (diffuse, moderate) |
| Liver | WNL | WNL | WNL |
| Lung | WNL | WNL | WNL |
| Pancreas | WNL | WNL | WNL + mononuclear cell infiltrate in adipose tissue (diffuse) |
| Prostate | WNL | WNL | WNL |
| Salivary glands | WNL | WNL | WNL |
| Sciatic nerve | WNL | WNL | WNL |
| Seminal vesicle | WNL | WNL | WNL |
| Skeletal muscle | WNL | WNL | WNL |
| Skin | WNL | WNL | WNL |
| Spleen | WNL | WNL + prominent extramedullary hematopoiesis | WNL + prominent extramedullary hematopoiesis |
| Stomach | WNL | WNL | WNL |
| Teste | WNL | WNL | WNL |
| Tibia (bone with bone marrow) | WNL | WNL | WNL |
| Trachea | WNL | WNL | WNL |

**Notes**: i.p.: intraperitoneal; WNL: within normal limits.

**Table S1. Toxicological analysis of WT mice chronically treated with 10 mg/kg of DN13-DN1, DN1 or with vehicle.** Summary of toxicological parameters assessed in wild-type (WT) mice chronically treated with 10 mg/kg of DN13-DN1, 10 mg/kg of DN1 nanobodies, or vehicle.
